# Stress-Rupture of Fiber-Reinforced Ceramic-Matrix Composites with Stochastic Loading at Intermediate Temperatures. Part I: Theoretical Analysis

**DOI:** 10.3390/ma12193123

**Published:** 2019-09-25

**Authors:** Longbiao Li

**Affiliations:** College of Civil Aviation, Nanjing University of Aeronautics and Astronautics, Nanjing 210016, China; llb451@nuaa.edu.cn; Tel.: +86-25-84895963

**Keywords:** ceramic-matrix composites (CMCs), stress-rupture, stochastic loading, matrix cracking, interface debonding, fiber failure, lifetime

## Abstract

Under stress-rupture loading, stochastic loading affects the internal damage evolution and lifetime of fiber-reinforced ceramic-matrix composites (CMCs) at intermediate temperatures. The damage mechanisms of the matrix cracking, fiber/matrix interface debonding and oxidation, and fiber fracture are considered in the analysis of stochastic loading. The strain, fiber/matrix interface debonding and oxidation length, and the broken fibers fraction versus the time curves of SiC/SiC composite under constant and three different stochastic loading conditions are analyzed. The effects of the stochastic loading stress level, stochastic loading time, and time spacing on the damage evolution and lifetime of SiC/SiC composite are discussed. When the stochastic loading stress level increases, the stress-rupture lifetime decreases, and the time for the interface complete debonding and oxidation decreases. When the stochastic loading time and time spacing increase, the stress-rupture lifetime decreases, and the time for the interface complete debonding and oxidation remains the same.

## 1. Introduction

Ceramic-matrix composites (CMCs) are a new type of thermal–structural–functional integrated material with the advantages of metal materials, ceramic materials, and carbon materials [1]. They have the characteristics of material–structural integration. Through the optimization design of each structural unit, synergistic effects can be produced, and high performance and reasonable matching of each performance can be achieved. Therefore, CMCs have high temperature resistance, corrosion resistance, wear resistance, low density, high specific strength, high specific modulus, low thermal expansion coefficient, insensitivity to cracks, no catastrophic damage, and other advantages [2]. Compared to metallic alloys, CMCs can have a density reduction of 30–50% and can exceed the working temperature range [3]. With the increase of thrust–weight ratio and turbine inlet temperature, CMCs have become one of the preferred high-temperature structural materials for aeroengines. When CMCs are used in hot-section components in aeroengines, i.e., turbine, combustion chamber, combustion liner, and nozzles, the amount of cooling air can be significantly reduced or even zero, the combustion efficiency can be improved, and the pollution emission and noise level can be reduced. At present, the application of CMCs in aeroengines follows the development idea from stationary parts to rotating parts, from intermediate temperature parts (i.e., 700–1000 °C) to high temperature parts (i.e., 1000–1300 °C), and gives priority to developing intermediate temperature and intermediate load (i.e., less than 120 MPa) stationary parts (i.e., seals and flaps, etc.), then the high temperature intermediate load (i.e., less than 120 MPa) stationary parts (i.e., flame tube, flame holder, turbine outer ring, guide vane, etc.), and then the high temperature and high load (i.e., higher than 120 MPa) rotating parts (i.e., turbine rotor, turbine blade, etc.). The CMC nozzle flaps, and seals have already been applied in M53-2, M88, M88-2, F100, F119, EJ200, F414, F110, and F136 aeroengines [4].

Since the applications for fiber-reinforced CMCs involve components with lives that are measured in tens of thousands of hours, the successful design and implementation of CMC components depend on the knowledge of the material behavior over periods of time comparable to the expected service life of the component [5]. In order to ensure the reliability and safety of fiber-reinforced CMCs hot-section components used in aeroengines, it is necessary to develop performance evaluation, damage evolution, and strength and life prediction tools or models [6,7,8]. Under constant stress loading at intermediate temperatures, multiple damage mechanisms of matrix cracking, fiber/matrix interface debonding, and interphase and fiber oxidation occurs in CMCs [9,10,11]. Hussain et al. [12] and Khosravani et al. [13] performed investigations on the thermal issues on composites. Morscher et al. [14] investigated the stress-rupture of a woven SiC/SiC composite with the BN interphase. Two regimes exist in the stress-rupture lifetime curve, i.e., a high-stress regime where rupture occurs at a fast rate and a low-stress regime where rupture occurs at a slower rate. Morscher and Cawley [15] investigated the time-dependent strength degradation of SiC/SiC composite at intermediate temperature. Li [16,17] investigated the damage evolution of cross-ply CMCs under stress-rupture and cyclic loading at elevated temperature. Momon et al. [18] and Godin et al. [19] investigated the stress-rupture lifetime of SiC/SiC composite using acoustic emission analysis. Ikarashi et al. [20] investigated the effect of cyclic tensile loading on the rupture behavior of orthogonal three-dimensional (3D) SiC/SiC composite at elevated temperature in air atmosphere. The matrix cracking propagation caused by the oxidation of the fiber/matrix interface and the degradation of the interfacial shear stress affects the lifetime of SiC/SiC composite. However, the effect of stochastic loading on the damage evolution and lifetime of CMCs has not been investigated.

The objective of this paper was to investigate the damage evolution and lifetime of fiber-reinforced CMCs under stress-rupture with stochastic loading at intermediate temperatures. Four different loading cases, including constant loading and stochastic loading with different stress levels, time, and time spacing, were considered in the analysis. The relationships between the stochastic loading stress, time and time spacing, the fiber/matrix interface debonding, broken fiber fraction, and lifetime of fiber-reinforced CMCs were established. The evolution of the strain, the fiber/matrix interface debonding and oxidation length, the broken fiber fraction, and the lifetime of SiC/SiC composite at 800 °C in air atmosphere was analyzed.

## 2. Theoretical model

When stochastic loading occurs during constant stress loading at an elevated temperature, the damage extent inside of fiber-reinforced CMCs becomes much more serious. In the present analysis, the shear-lag model was used to analyze the stress distribution of damaged CMCs under stress-rupture with stochastic loading. The damage mechanisms of the matrix cracking, fiber/matrix interface debonding and oxidation, and broken fibers were considered. The matrix stochastic cracking model, fracture mechanics approach, and Global Load Sharing criterion were used to determine the matrix crack spacing, fiber/matrix interface debonding length, and the broken fibers fraction under stress-rupture with stochastic loading. The constitutive relationship considering the time-dependent damage mechanisms was also developed.

**Figure 1:**
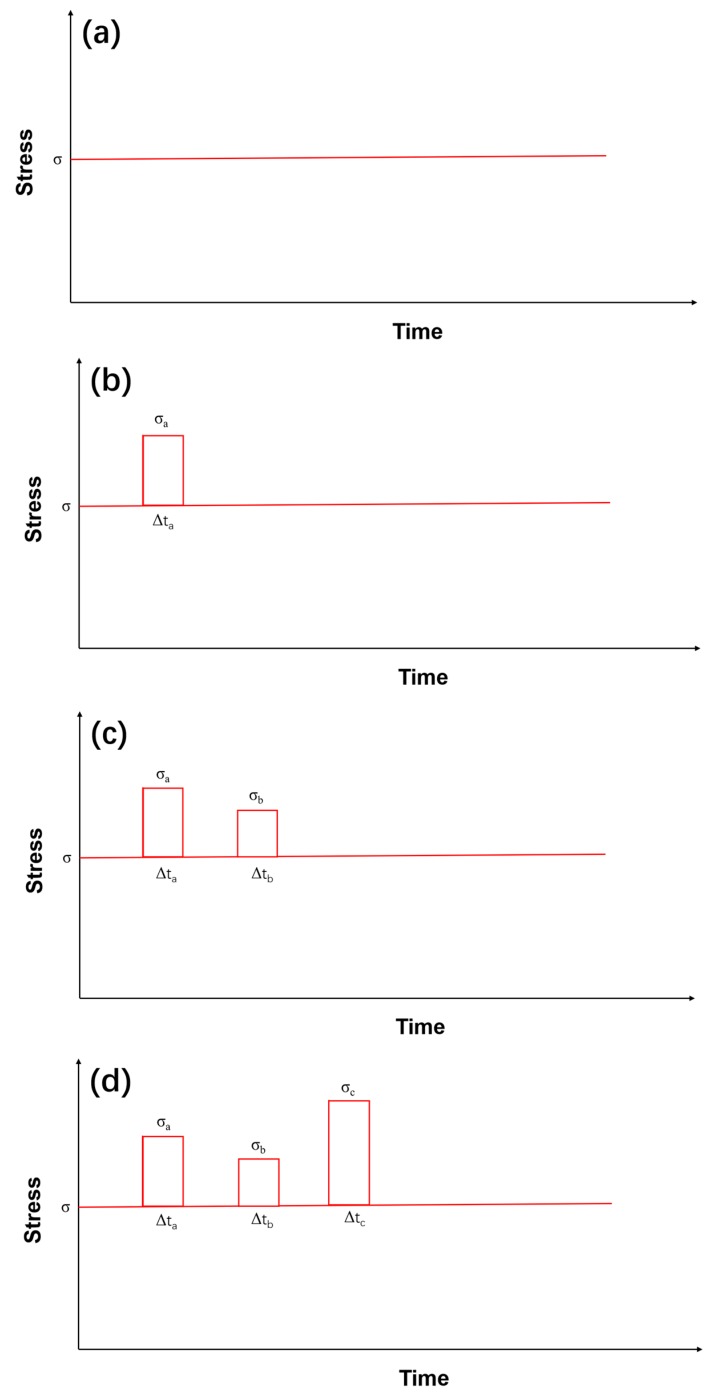
The schematic of loading sequence of (**a**) constant loading; (**b**) stochastic loading of *σ*_a_ and Δ*t*_a_; (**c**) stochastic loading of *σ*_a_, *σ*_b_ and Δ*t*_a_, Δ*t*_b_; and (**d**) stochastic loading of *σ*_a_, *σ*_b_, *σ*_c_ and Δ*t*_a_, Δ*t*_b_, Δ*t*_c_.

Figure 1 shows the stochasic loading sequence under constant stress-rupture laoding of fiber-reinforced CMCs at an elevated temperatrue, which can be divided into four cases, as follows:

(1) Case I, constant stress loading;
(2) Case II, constant stress loading and stochsatic loading of *σ*_a_ with Δ*t*_a_;
(3) Case III, constant stress loading and stochastic loading of *σ*_a_ and *σ*_b_ with Δ*t*_a_ and Δ*t*_b_;
(4) Case IV, constant stress loading and stochastic loading of *σ*_a_, *σ*_b_ and *σ*_c_ with Δ*t*_a_, Δ*t*_b_, and Δ*t*_c_.

**Figure 2:**
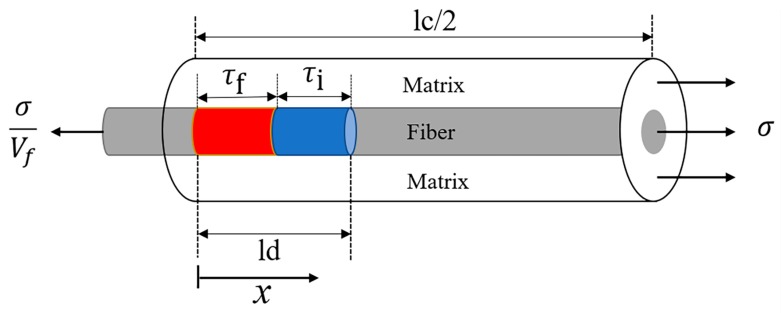
The unit cell of the shear-lag model.

Figure 2 shows a unit cell used for the stress analysis of the fiber and the matrix when the matrix cracking, fiber/matrix interface debonding, and fiber failure appear inside of CMCs. When the fiber fractures under stochastic loading, the fiber axial stress distribution can be determined using the following equation:

(1) $$\sigma_{f}\left(x,t\right)=\left\{ \begin{array}{l} T_{S}\left(t\right)-\frac{2\tau_{f}}{r_{f}}x,x\in \left[0,\zeta \left(t\right)\right]\\ T_{S}\left(t\right)-\frac{2\tau_{f}}{r_{f}}\zeta \left(t\right)-\frac{2\tau_{i}}{r_{f}}\left(x-\zeta \left(t\right)\right),x\in \left[\zeta \left(t\right),l_{d}\left(t\right)\right]\\ \sigma_{\mathrm{fo}}+\left[T_{S}\left(t\right)-\sigma_{\mathrm{fo}}-\frac{2\tau_{f}}{r_{f}}\zeta \left(t\right)-\frac{2\tau_{i}}{r_{f}}\left(l_{d}\left(t\right)-\zeta \left(t\right)\right)\right]\exp\left(-\rho \frac{x-l_{d}\left(t\right)}{r_{f}}\right),x\in \left[l_{d}\left(t\right),\frac{l_{c}}{2}\right] \end{array}\right.$$

where *r*_f_ denotes the fiber radius; *τ*_f_ denotes the fiber/matrix interface shear stress in the oxidation region; *τ*_i_ denotes the fiber/matrix interface shear stress in the slip region; *T_S_*(*t*) denotes the intact fiber stress under stochastic loading; *l*_d_(*t*) denotes the time-dependent fiber/matrix interface debonding length under stochastic loading; *l*_c_ denotes the matrix crack spacing under stochastic loading; *ρ* denotes the shear-lag model parameter; and *ζ*(*t*) denotes the time-dependent fiber/matrix interface oxidation length [21].

(2) $$\zeta \left(t\right)=\varphi_{1}\left[1-\exp\left(-\frac{\varphi_{2}t}{b}\right)\right]$$

where *b* is a delay factor considering the deceleration of reduced oxygen activity, and *φ*_1_ and *φ*_2_ are parameters dependent on temperature and described using the Arrhenius type laws. The fiber axial stress in the fiber/matrix interface bonded region can be determined using the following equation:

(3) $$\sigma_{\mathrm{fo}}=\frac{E_{f}}{E_{c}}\sigma +E_{f}\left(\alpha_{c}-\alpha_{f}\right)\Delta T$$

where *E*_f_, and *E*_c_ denote the fiber and the composite elastic modulus, respectively; *α*_f_, and *α*_c_ denote the fiber and the composite thermal expansion coefficient, respectively; and ΔT denotes the temepratrue difference between the testing temperature and the fabrication temperatrue.

The matrix cracking under stochastic loading can be described using the two-parameter Weibull distribution, and the time-dependent matrix crack spacing under stochastic loading can be determined using the following equation [22]:

(4) $$l_{c}=r_{f}\frac{V_{m}E_{m}}{V_{f}E_{c}}\frac{\sigma_{R}}{2\tau_{i}}\Lambda {\left\{1-\exp\left[-{\left(\frac{\sigma_{S}-\left(\sigma_{\mathrm{mc}}-\sigma_{\mathrm{th}}\right)}{\left(\sigma_{R}-\sigma_{\mathrm{th}}\right)-\left(\sigma_{\mathrm{mc}}-\sigma_{\mathrm{th}}\right)}\right)}^{m}\right]\right\}}^{-1}$$

where *σ_S_* denotes the stochastic loading stress; *E*_m_ denotes the matrix elastic modulus; *σ*_R_ denotes the matrix cracking characteristic strength; *σ*_mc_ denotes matrix first cracking stress; *σ*_th_ denotes matrix thermal residual stress; Λ denotes the final nominal crack space; and *m* denotes matrix Weibull modulus.

The time-dependent fiber/matrix interface debonding length under stochastic loading can be determined using the fracture mechanics approach [23]:

(5) $$\xi_{d}=-\frac{F}{4\pi r_{f}}\frac{\partial w_{f}\left(\sigma_{S},t\right)}{\partial l_{d}}-\frac{1}{2}\int_{0}^{l_{d}}\tau_{i}\frac{\partial v\left(\sigma_{S},t\right)}{\partial l_{d}}dx$$

where *ξ*_d_ denotes the fiber/matrix interface debonding energy; *F*(= π*r*_f_^2^*σ*/*V*_f_) denotes the fiber stress at the matrix cracking plane; *w*_f_ (*σ_S_*, *t*) denotes the time-dependent fiber axial displacement under stochastic loading at the matrix cracking plane; and *v*(*σ_S_*, *t*) denotes the time-dependent relative displacement between the fiber and the matrix under stochastic loading. Substituting the time-dependent fiber axial displacement and relative displacement into Equation (5), the time-dependent fiber/matrix interface debonding length under stochastic loading can be determined using the following equation:

(6) $$l_{d}\left(\sigma_{S},t\right)=\left(1-\eta \right)\zeta \left(t\right)+\frac{r_{f}}{2}\left(\frac{V_{m}E_{m}T_{S}}{E_{c}\tau_{i}}-\frac{1}{\rho }\right)-\sqrt{{\left(\frac{r_{f}}{2\rho }\right)}^{2}-\frac{r_{f}^{2}V_{f}V_{m}E_{f}E_{m}T_{S}^{2}}{4E_{c}^{2}\tau_{i}^{2}}\left(1-\frac{\sigma }{V_{f}T_{S}}\right)+\frac{r_{f}V_{m}E_{f}E_{m}}{E_{c}\tau_{i}^{2}}\xi_{d}}$$

where *η* = *τ*_f_/*τ*_i_.

The two-parameter Weibull model was adopted to describe the fiber strength distribution, and the Global Load Sharing criterion was used to determine the stress distributions between the intact and fracture fibers [24].

(7) $$\frac{\sigma }{V_{f}}=T_{S}\left(1-P\left(T_{S}\right)\right)+\frac{2\tau_{f}}{r_{f}}\langle L\rangle P\left(T_{S}\right)$$

where 〈L〉 denotes the average fiber pullout length, and *P*(*T_S_*) denotes the fiber failure probability.

(8) $$P\left(T_{S}\right)=1-\exp\left[-{\left(\frac{T_{S}}{\sigma_{c}}\right)}^{m_{f}+1}\right]$$

where *m*_f_ denotes the fiber Weibull modulus, and *σ*_c_ denotes the fiber characteristic strength of a length *δ*_c_ of fiber.

(9) $$\sigma_{c}={\left(\frac{l_{o}\sigma_{0}^{m_{f}}\left(t\right)\tau_{i}}{r_{f}}\right)}^{\frac{1}{m_{f}+1}},\delta_{c}={\left(\frac{\sigma_{0}\left(t\right)r_{f}l_{o}^{\frac{1}{m_{f}}}}{\tau_{i}}\right)}^{\frac{m_{f}}{m_{f}+1}}$$

where [25].

(10) $$\sigma_{0}\left(t\right)=\left\{ \begin{array}{l} \sigma_{0},t\leq \frac{1}{k}{\left(\frac{K_{\mathrm{IC}}}{Y\sigma_{0}}\right)}^{4}\\ \frac{K_{\mathrm{IC}}}{Y\sqrt[{4}]{kt}},t>\frac{1}{k}{\left(\frac{K_{\mathrm{IC}}}{Y\sigma_{0}}\right)}^{4} \end{array}\right.$$

where *σ*_0_ denotes the time-dependent fiber strength; *K*_IC_ denotes the fracture toughness; *Y* denotes the geometric parameter; and *k* is the parabolic rate constant.

When multiple damage mechanisms form inside of fiber-reinforced CMCs, the average composite strain of *ε*_c_(*t*) can be determined by integration of the axial strain in the fiber.

(11) $$\varepsilon_{c}\left(\sigma_{S},t\right)=\frac{2}{E_{f}l_{c}}\int_{l_{c}/2}\sigma_{f}\left(x,t\right)dx-\left(\alpha_{c}-\alpha_{f}\right)\Delta T$$

Substituting the time-dependent fiber axial stress under stochastic loading in Equation (1) into Equation (11), the composite average strain of *ε*_c_(*σ_S_*, *t*) can be determined using the following equation:

(12) $$\varepsilon_{c}\left(\sigma_{S},t\right)=\left\{ \begin{array}{l} \frac{T_{S}\left(t\right)}{E_{f}}\frac{2l_{d}\left(t\right)}{l_{c}}+\frac{2\tau_{f}}{r_{f}E_{f}l_{c}}\zeta^{2}\left(t\right)-\frac{4\tau_{f}l_{d}\left(t\right)}{r_{f}E_{f}l_{c}}\zeta \left(t\right)-\frac{2\tau_{i}}{r_{f}E_{f}l_{c}}{\left(l_{d}\left(t\right)-\zeta \left(t\right)\right)}^{2}+\frac{2\sigma_{\mathrm{fo}}}{E_{f}l_{c}}\left(\frac{l_{c}}{2}-l_{d}\left(t\right)\right)\\ +\frac{2r_{f}}{\rho E_{f}l_{c}}\left\{T_{S}\left(t\right)-\frac{2\tau_{f}}{r_{f}}\zeta \left(t\right)-\frac{2\tau_{i}}{r_{f}}\left[l_{d}\left(t\right)-\zeta \left(t\right)\right]-\sigma_{\mathrm{fo}}\right\}\\ \times \left[1-\exp\left(-\rho \frac{l_{c}/2-l_{d}\left(t\right)}{r_{f}}\right)\right]-\left(\alpha_{c}-\alpha_{f}\right)\Delta T,l_{d}\left(t\right)<\frac{l_{c}}{2}\\ \frac{T_{S}\left(t\right)}{E_{f}}\frac{2l_{d}\left(t\right)}{l_{c}}+\frac{2\tau_{f}}{r_{f}E_{f}l_{c}}\zeta^{2}\left(t\right)-\frac{4\tau_{f}l_{d}\left(t\right)}{r_{f}E_{f}l_{c}}\zeta \left(t\right)-\frac{2\tau_{i}}{r_{f}E_{f}l_{c}}{\left(l_{d}\left(t\right)-\zeta \left(t\right)\right)}^{2},l_{d}\left(t\right)=\frac{l_{c}}{2} \end{array}\right.$$

## 3. Results and analysis

The strain, fiber/matrix interface debonding and oxidation length, and broken fibers fraction versus the time curves of SiC/SiC composite were analyzed for the Cases II, III, and IV. The material properties were given by: *V*_f_ = 20%, *E*_f_ = 270 GPa, *E*_m_ = 400 GPa, *r*_f_ = 7 μm, *m* = 3, *α*_f_ =3.5 × 10^−6^/°C, *α*_m_ = 4.6 × 10^−6^/°C, ∆T = −1000 °C, *ξ*_d_ = 0.1 J/m^2^, *τ*_i_ = 30 MPa, *τ*_f_ = 1 MPa, *σ*_0_ = 2.5 GPa, *l*_0_ = 25 mm, *m*_f_ = 5, and Tem = 800 °C.

### 3.1. Case II

**Figure 3:**
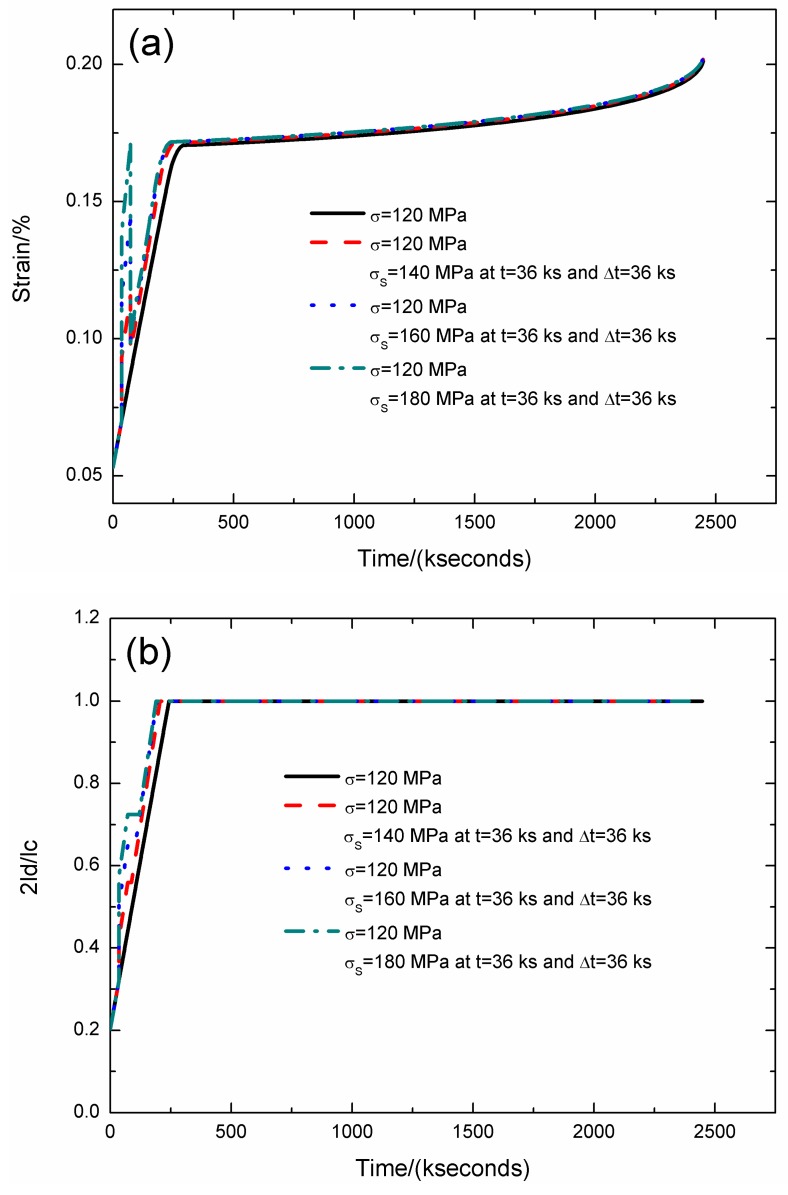

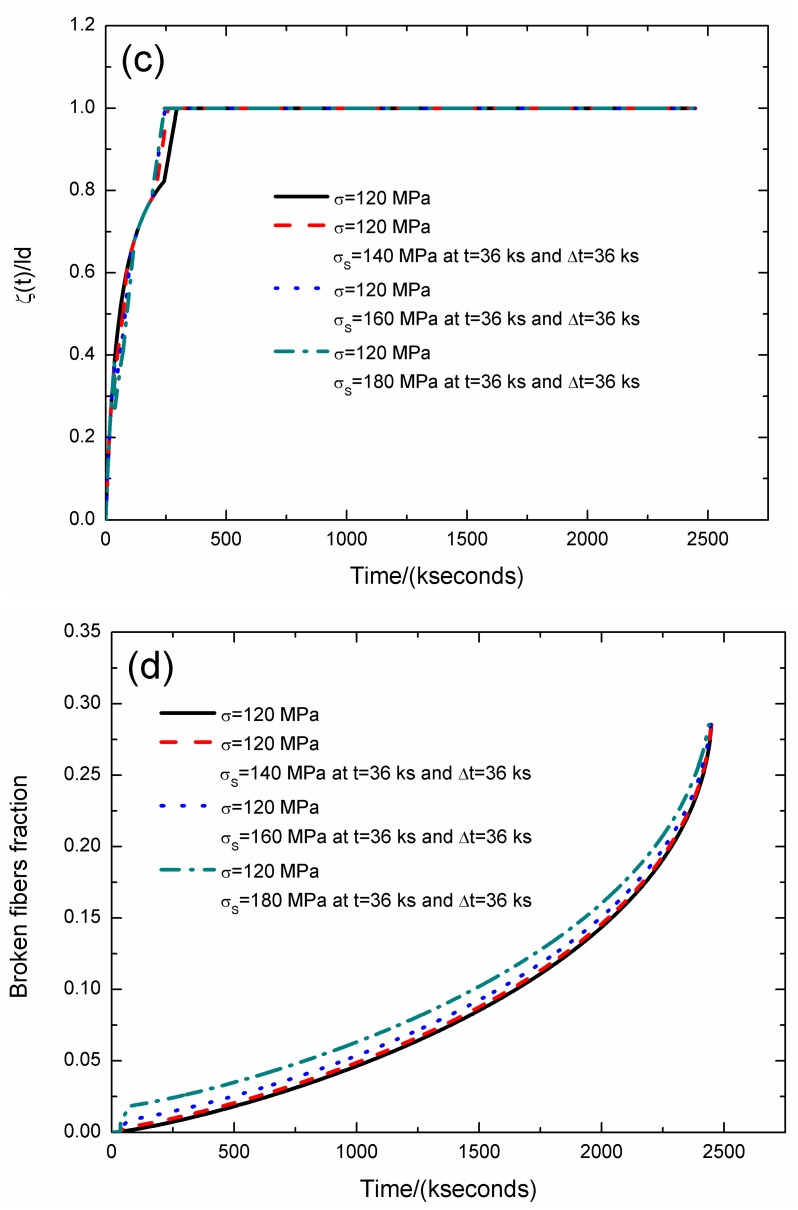
(**a**) The strain versus the time curves; (**b**) the fiber/matrix interface debonding length versus the time curves; (**c**) the fiber/matrix interface oxidation length versus the time curves; and (**d**) the broken fibers fraction versus the time curves of SiC/SiC composite under stress-rupture loading of constant stress of *σ* = 120 MPa, *σ*_S_ = 140, 160, 180 MPa at *t* = 36 kseconds and Δt = 36 kseconds at 800 °C in air atmosphere.

For the stochastic loading of Case II, the strain, interface debonding and oxidation length, and the broken fibers fraction of SiC/SiC composite under stress-rupture loading of constant stress of *σ* = 120 MPa, *σ*_S_ = 140, 160, and 180 MPa at *t* = 36 kseconds and Δ*t* = 36 kseconds at 800 °C in air atmosphere are shown in Figure 3 and Table 1. When the stochastic loading stress level increases, the stress-rupture lifetime decreases, and the time for the interface complete debonding and oxidation decreases.

Under constant stress loading of *σ* = 120 MPa, the stress-rupture lifetime is *t* = 2447.9 kseconds; the time for the interface complete debonding is *t* = 242.7 kseconds; the time for the interface complete oxidation is *t* = 295.3 kseconds; the failure strain is *ε*_c_ = 0.201%; and the broken fibers fraction is *P* = 0.285. When the stochastic loading stress is *σ*_S_ = 140 MPa, the stress-rupture lifetime is *t* = 2446.9 kseconds; the time for the interface complete debonding is *t* = 205.7 kseconds; the time for the interface complete oxidation is *t* = 259.4 kseconds; the failure strain is *ε*_c_ = 0.202%; and the broken fibers fraction is *P* = 0.285. When the stochastic loading stress is *σ*_S_ = 160 MPa, the stress-rupture lifetime is *t* = 2444.3 kseconds; the time for the interface complete debonding is *t* = 192.6 kseconds; the time for the interface complete oxidation is *t* = 246.8 kseconds; the failure strain is *ε*_c_ = 0.201%; and the broken fibers fraction is *P* = 0.285. When the stochastic loading stress is *σ*_S_ = 180 MPa, the stress-rupture lifetime is *t* = 2437 kseconds; the time for the interface complete debonding is *t* = 189.1 kseconds; the time for the interface complete oxidation is *t* = 243.1 kseconds; the failure strain is *ε*_c_ = 0.2%; and the broken fibers fraction is *P* = 0.285.

**Figure 4:**
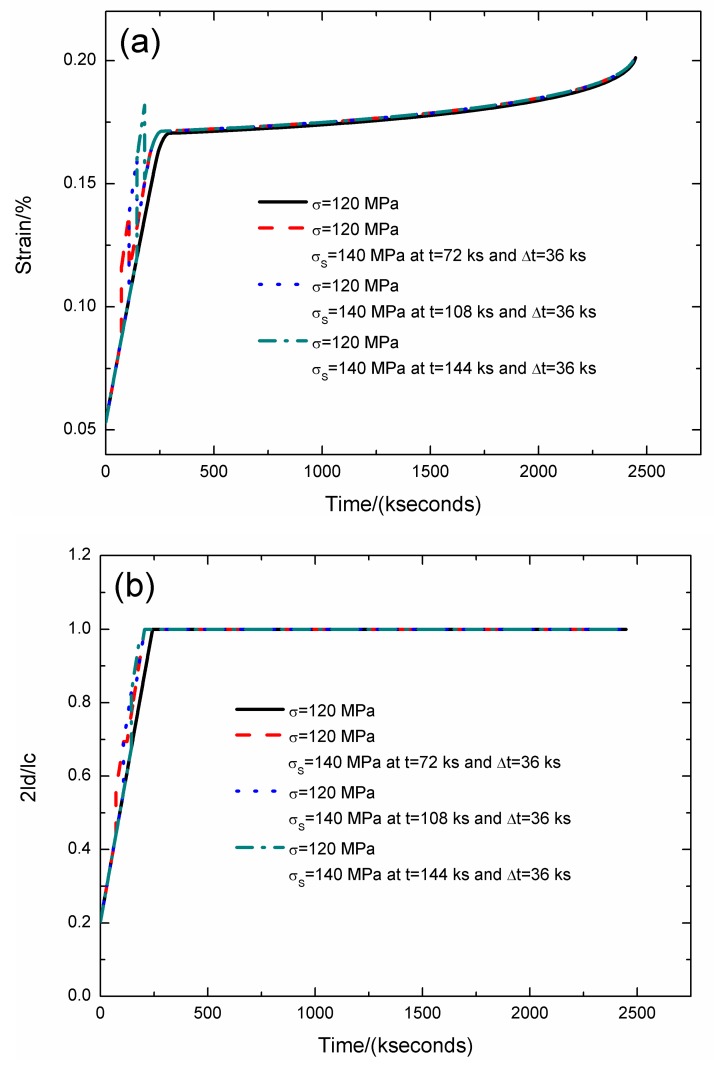

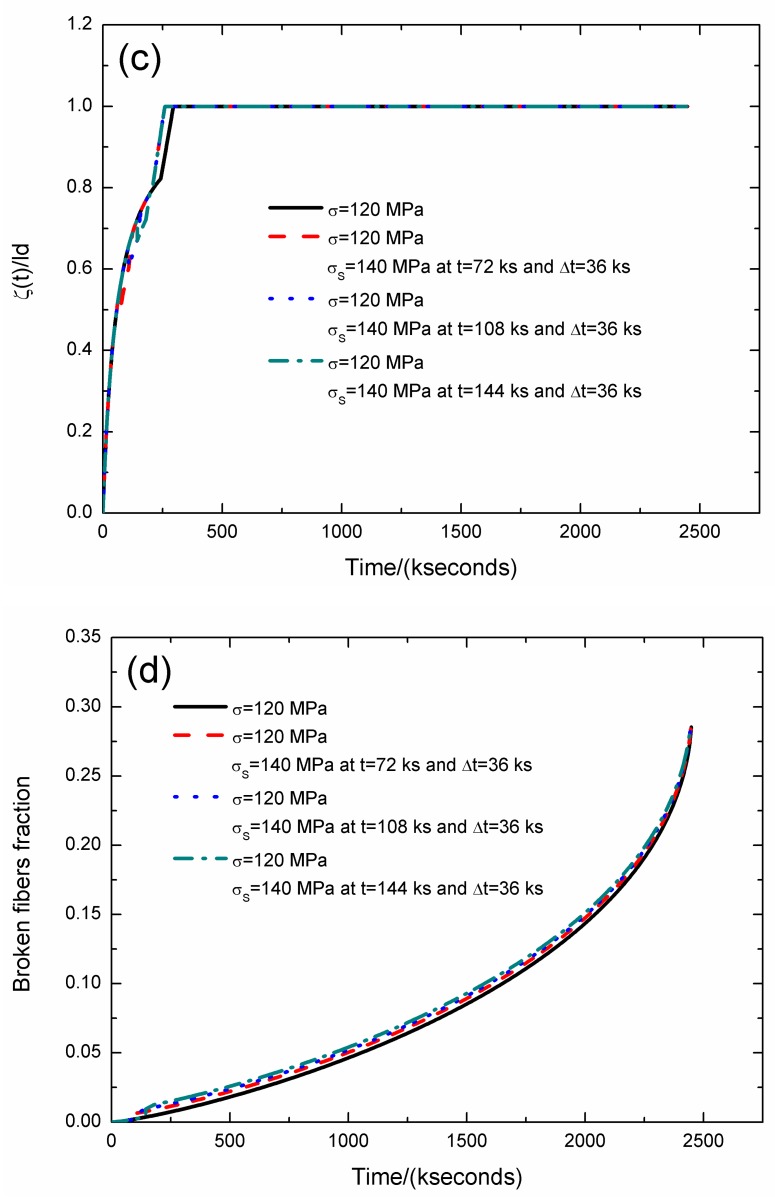
(**a**) The strain versus the time curves; (**b**) the fiber/matrix interface debonding length versus the time curves; (**c**) the fiber/matrix interface oxidation length versus the time curves; and (**d**) the broken fibers fraction versus the time curves of SiC/SiC composite under stress-rupture loading of constant stress of *σ* = 120 MPa, *σ*_S_ = 140 MPa at *t* = 72, 108, 144 kseconds and Δt = 26 kseconds at 800 °C in air atmosphere.

The strain, interface debonding and oxidation length, and the broken fibers fraction of SiC/SiC composite under stress-rupture loading of constant stress of *σ* = 120 MPa, *σ*_S_ = 140 MPa at *t* = 72, 108, 144 kseconds and Δ*t* = 36 kseconds at 800 °C in air atmosphere are shown in Figure 4 and Table 2.

When the stochastic loading time is *t* = 72 kseconds, the stress-rupture lifetime is *t* = 2446.2 kseconds; the time for the interface complete debonding is *t* = 205.7 kseconds; the time for the interface complete oxidation is *t* = 259.4 kseconds; the failure strain is *ε*_c_ = 0.201%; and the broken fibers fraction is *P* = 0.285. When the stochastic loading time increases from *t* = 72 to 144 kseconds, the stress-rupture lifetime decreases, and the time for the interface complete debonding and oxidation remains the same.

**Figure 5:**
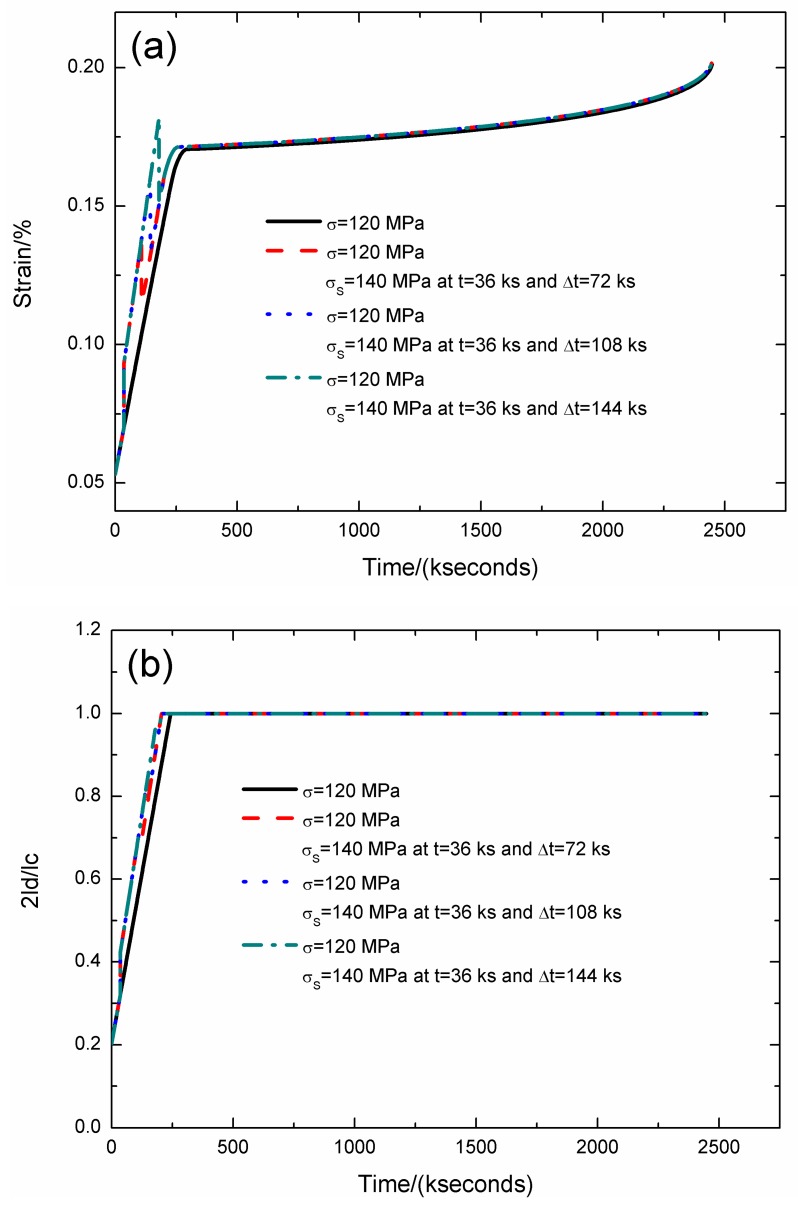

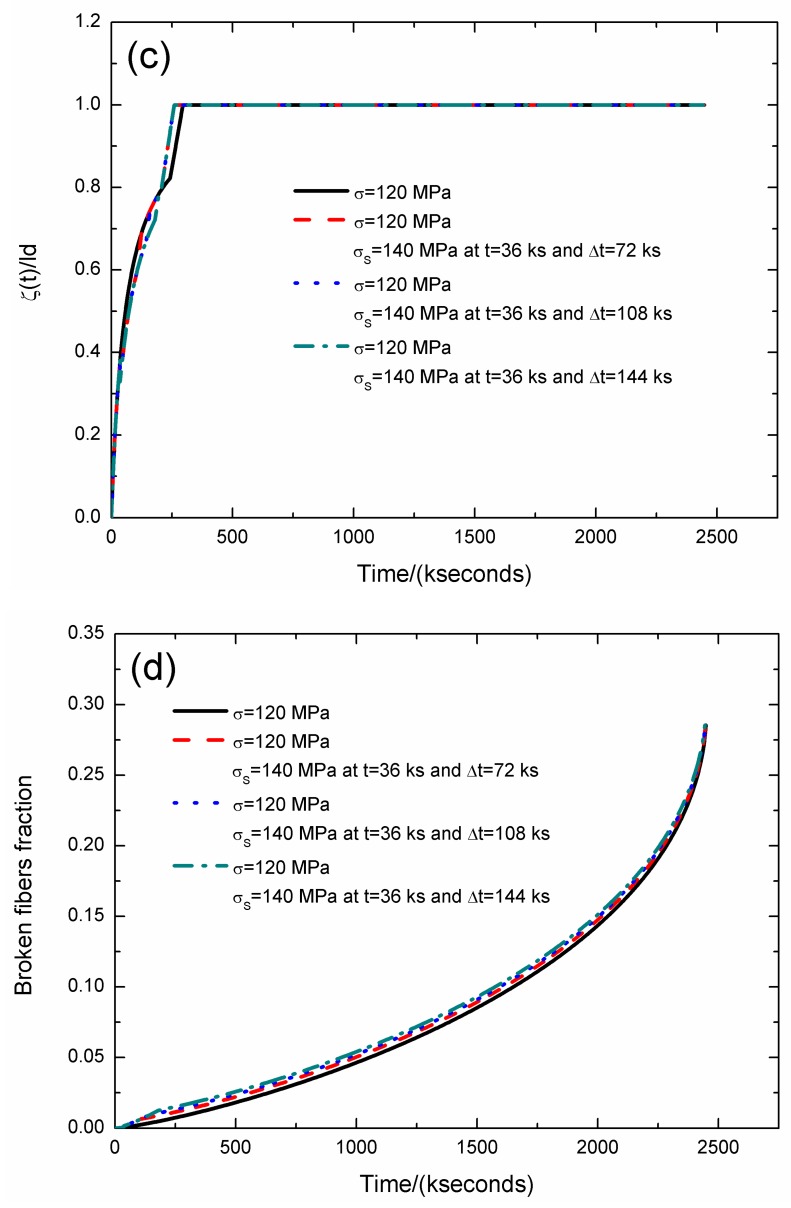
(**a**) The strain versus the time curves; (**b**) the fiber/matrix interface debonding length versus the time curves; (**c**) the fiber/matrix interface oxidation length versus the time curves; and (**d**) the broken fibers fraction versus the time curves of SiC/SiC composite under stress-rupture loading of constant stress of *σ* = 120 MPa, *σ*_S_ = 140 MPa at *t* = 36 kseconds and Δt = 72, 108, 144 kseconds at 800 °C in air atmosphere.

The strain, fiber/matrix interface debonding and oxidation length, and the broken fibers fraction of SiC/SiC composite under stress-rupture loading of constant stress of *σ* = 120 MPa, *σ*_S_ = 140 MPa at *t* = 36 kseconds and Δ*t* = 72, 108, 144 kseconds at 800 °C in air atmosphere are shown in Figure 5 and Table 3.

When the stochastic loading time spacing is Δ*t* = 72 kseconds, the stress-rupture lifetime is *t* = 2446.2 kseconds; the time for the interface complete debonding is *t* = 205.7 kseconds; the time for the interface complete oxidation is *t* = 259.4 kseconds; the failure strain is *ε*_c_ = 0.201%; and the broken fibers fraction is *P* = 0.285. When the stochastic loading time spacing increases from Δ*t* = 72 to 144 kseconds, the stress-rupture lifetime decreases, and the time for the interface complete debonding and oxidation remains the same.

### 3.2. Case III

**Figure 6:**
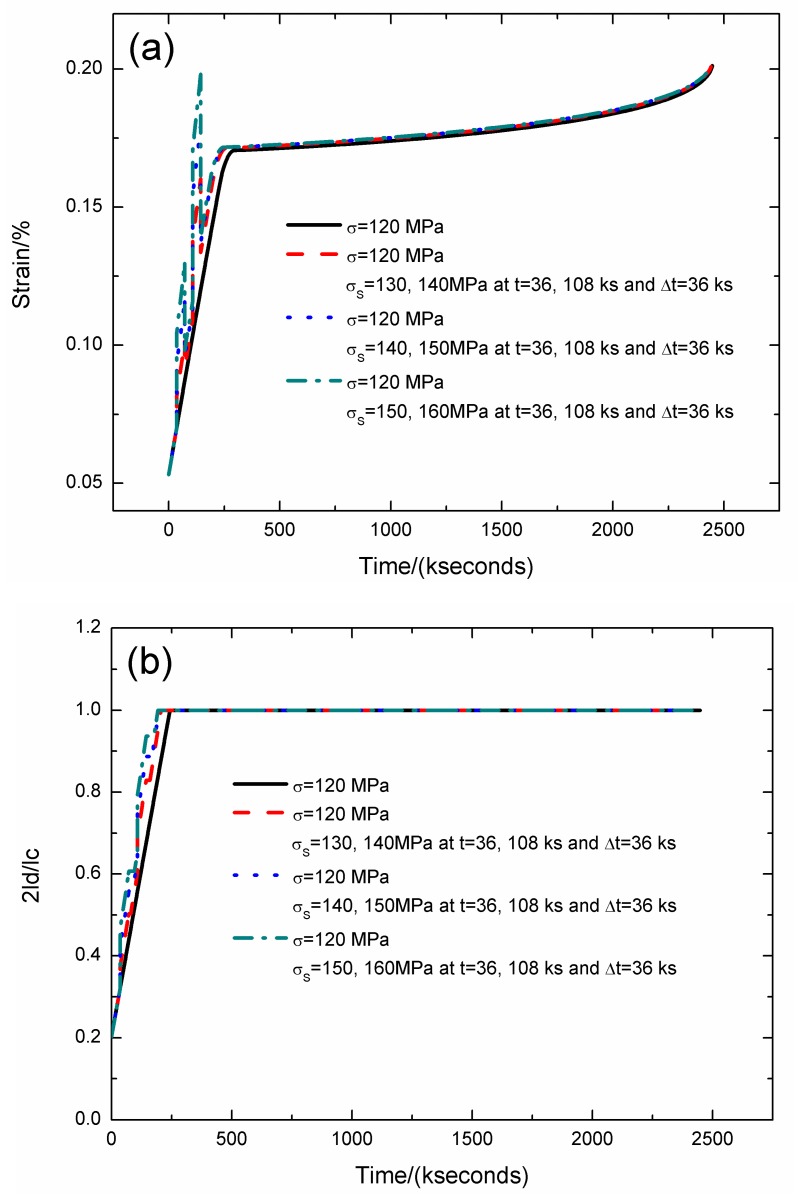

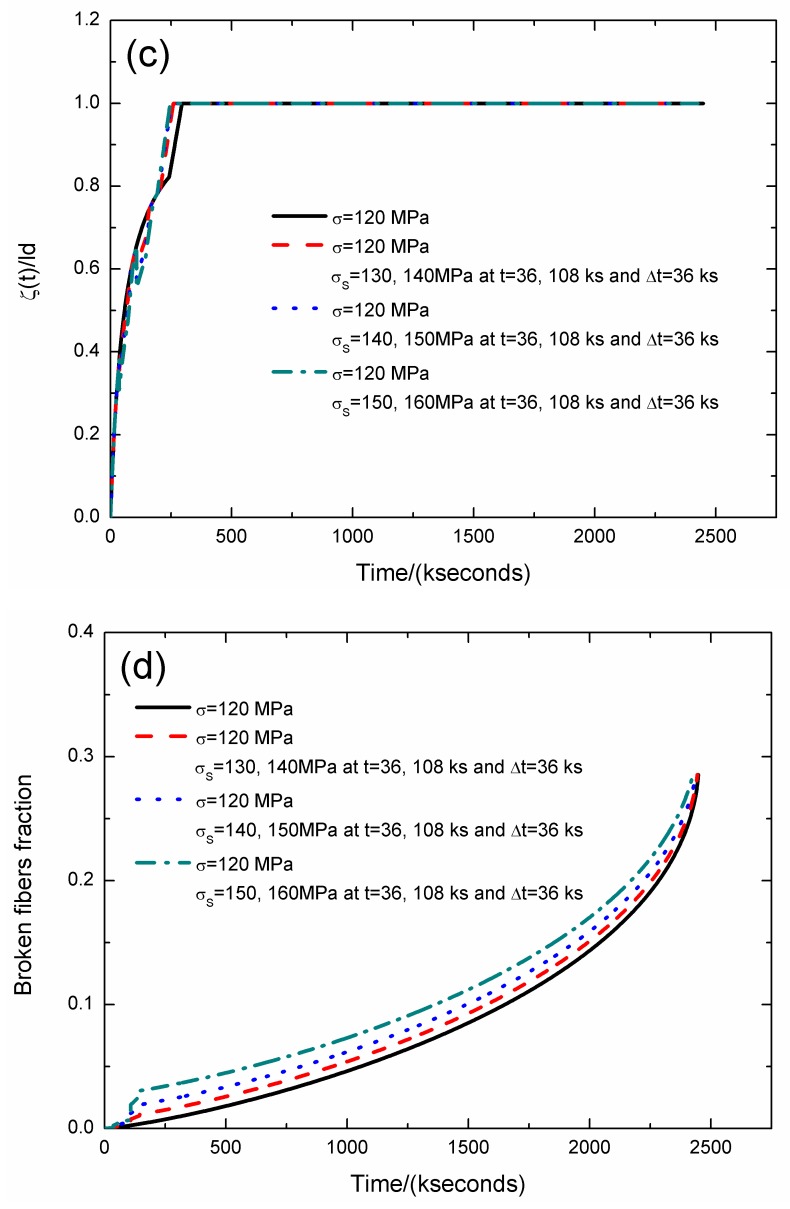
(**a**) The strain versus the time curves; (**b**) the fiber/matrix interface debonding length versus the time curves; (**c**) the fiber/matrix interface oxidation length versus the time curves; and (**d**) the broken fibers fraction versus the time curves of SiC/SiC composite under stress-rupture loading of constant stress of *σ* = 120 MPa, *σ*_S_ = 130/140, 140/150, 150/160 MPa at *t* = 36/108 kseconds and Δt = 36 kseconds at 800 °C in air atmosphere.

For the stochastic loading of Case III, the strain, fiber/matrix interface debonding and oxidation length, and the broken fibers fraction of SiC/SiC composite under stress-rupture loading of constant stress of *σ* = 120 MPa, *σ*_S_ = 130/140, 140/150, 150/160 MPa at *t* = 36/108 kseconds and Δ*t* = 36 kseconds at 800 °C in air atmosphere are shown in Figure 6 and Table 4. When the stochastic loading stress increases, the stress-rupture lifetime decreases, and the time for the interface complete debonding and oxidation decreases.

When the stochastic loading stress is *σ*_S_ = 130, 140 MPa, the stress-rupture lifetime is *t* = 2444.1 kseconds; the time for the interface complete debonding is *t* = 205.7 kseconds; the time for the interface complete oxidation is *t* = 259.4 kseconds; the failure strain is *ε*_c_ = 0.2%; and the broken fibers fraction is *P* = 0.285. When the stochastic loading stress is *σ*_S_ = 140, 150 MPa, the stress-rupture lifetime is *t* = 2438.1 kseconds; the time for the interface complete debonding is *t* = 197.3 kseconds; the time for the interface complete oxidation is *t* = 251.3 kseconds; the failure strain is *ε*_c_ = 0.2%; and the broken fibers fraction is *P* = 0.285. When the stochastic loading stress is *σ*_S_ = 150, 160 MPa, the stress-rupture lifetime is *t* = 2425.9 kseconds; the time for the interface complete debonding is *t* = 192.6 kseconds; the time for the interface complete oxidation is *t* = 246.8 kseconds; the failure strain is *ε*_c_ = 0.199%; and the broken fibers fraction is *P* = 0.285.

**Figure 7:**
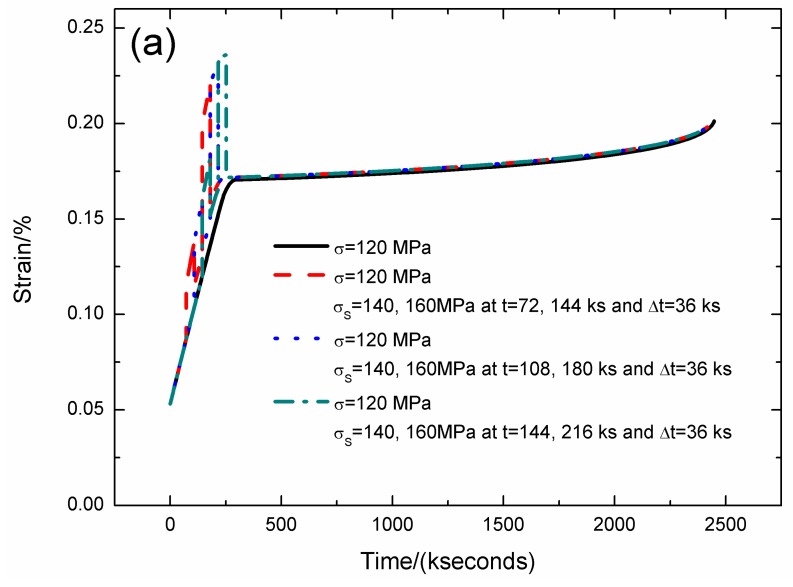

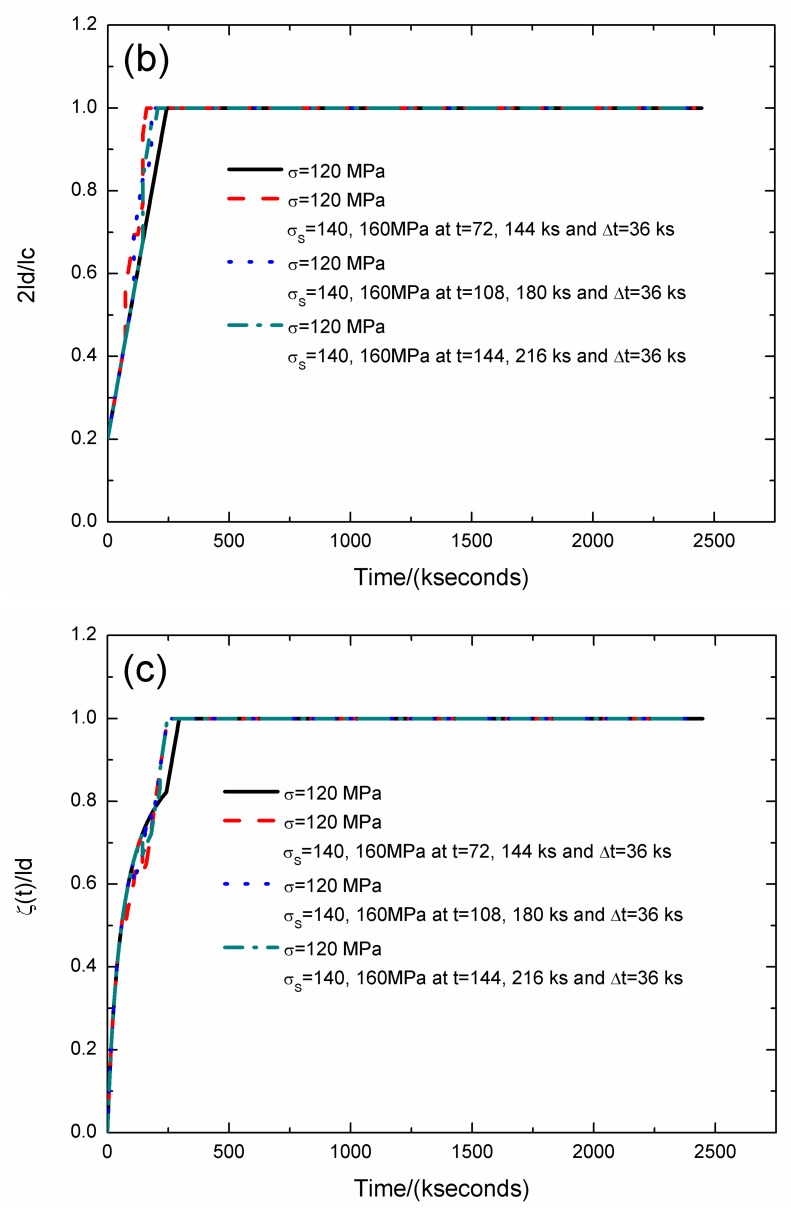

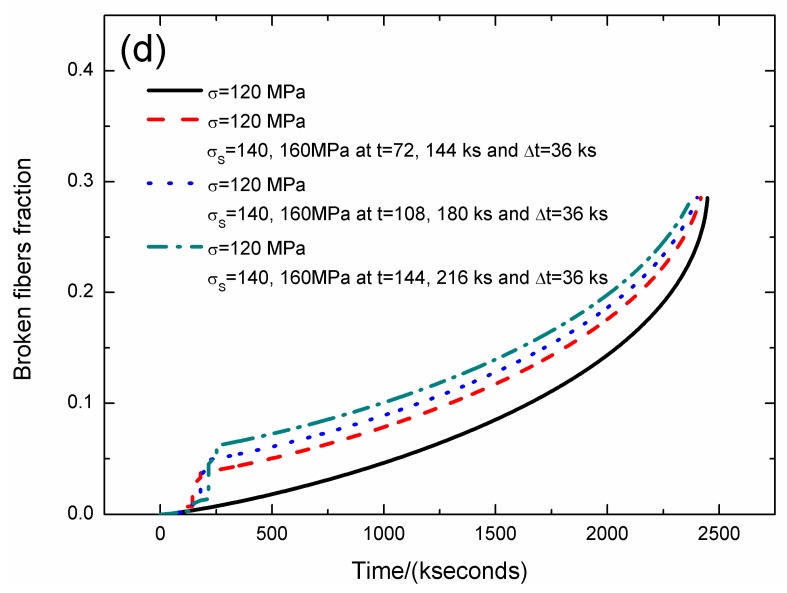
(**a**) The strain versus the time curves; (**b**) the fiber/matrix interface debonding length versus the time curves; (**c**) the fiber/matrix interface oxidation length versus the time curves; and (**d**) the broken fibers fraction versus the time curves of SiC/SiC composite under stress-rupture loading of constant stress of *σ* = 120 MPa, *σ*_S_ = 140/160MPa at *t* = 72/144, 108/180, 144/216 kseconds and Δt = 36 kseconds at 800 °C in air atmosphere.

The strain, fiber/matrix interface debonding and oxidation length, and the broken fibers fraction of SiC/SiC composite under stress-rupture loading of constant stress of *σ* = 120 MPa, *σ*_S_ = 140/160 MPa at *t* = 72/144, 108/180, 144/216 kseconds and Δ*t* = 36 kseconds at 800 °C in air atmosphere are shown in Figure 7 and Table 5. When the stochastic loading time increases, the stress-rupture lifetime decreases, and the time for the interface complete debonding increases.

When the stochastic loading time is *t* = 72 and 144 kseconds, the stress-rupture lifetime is *t* = 2418.4 kseconds; the time for the interface complete debonding is *t* = 160 kseconds at stochastic loading stress of *σ*_S_ = 160 MPa; the time for the interface complete oxidation is *t* = 246.8 kseconds at constant loading stress of *σ* = 120 MPa; the failure strain is *ε*_c_ = 0.198%; and the broken fibers fraction is *P* = 0.285. When the stochastic loading time is *t* = 144 and 216 kseconds, the stress-rupture lifetime is *t* = 2376.8 kseconds; the time for the interface complete debonding is *t* = 205.7 kseconds at constant stress of *σ* = 120 MPa; the time for the interface complete oxidation is *t* = 246.8 kseconds; the failure strain is *ε*_c_ = 0.195%; and the broken fibers fraction is *P* = 0.285.

**Figure 8:**
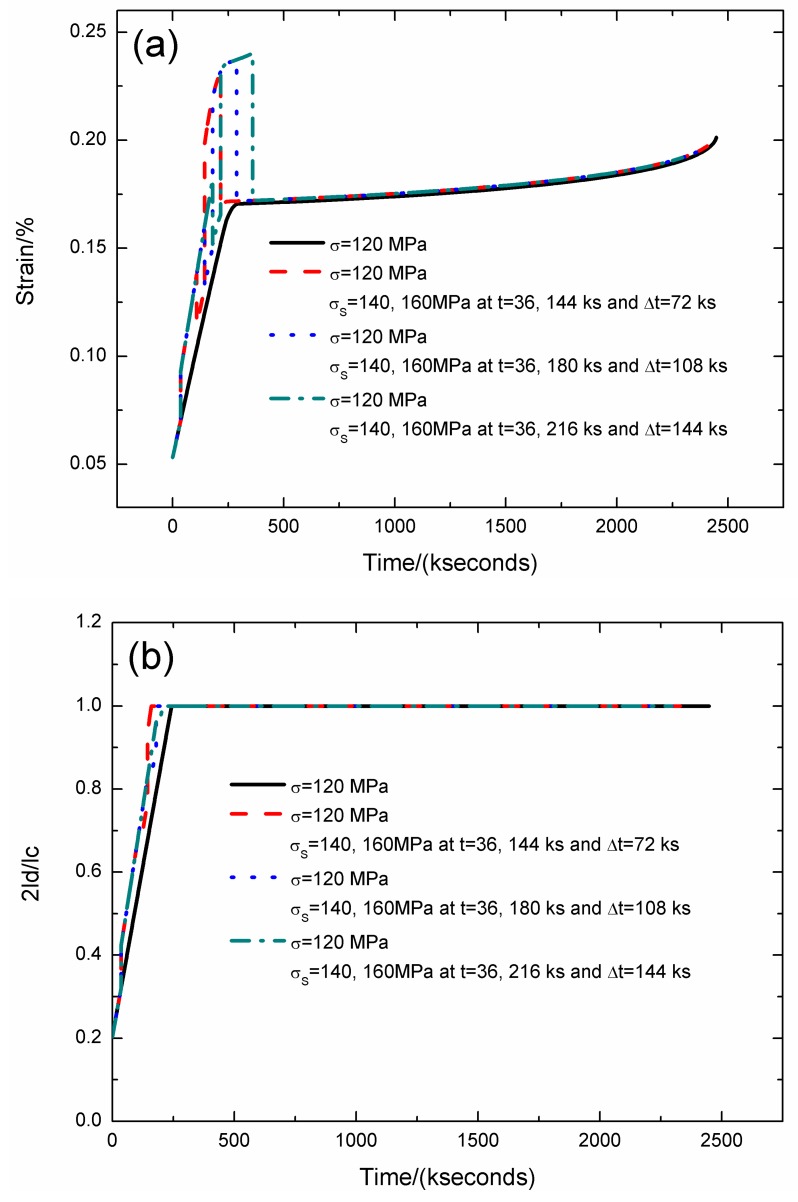

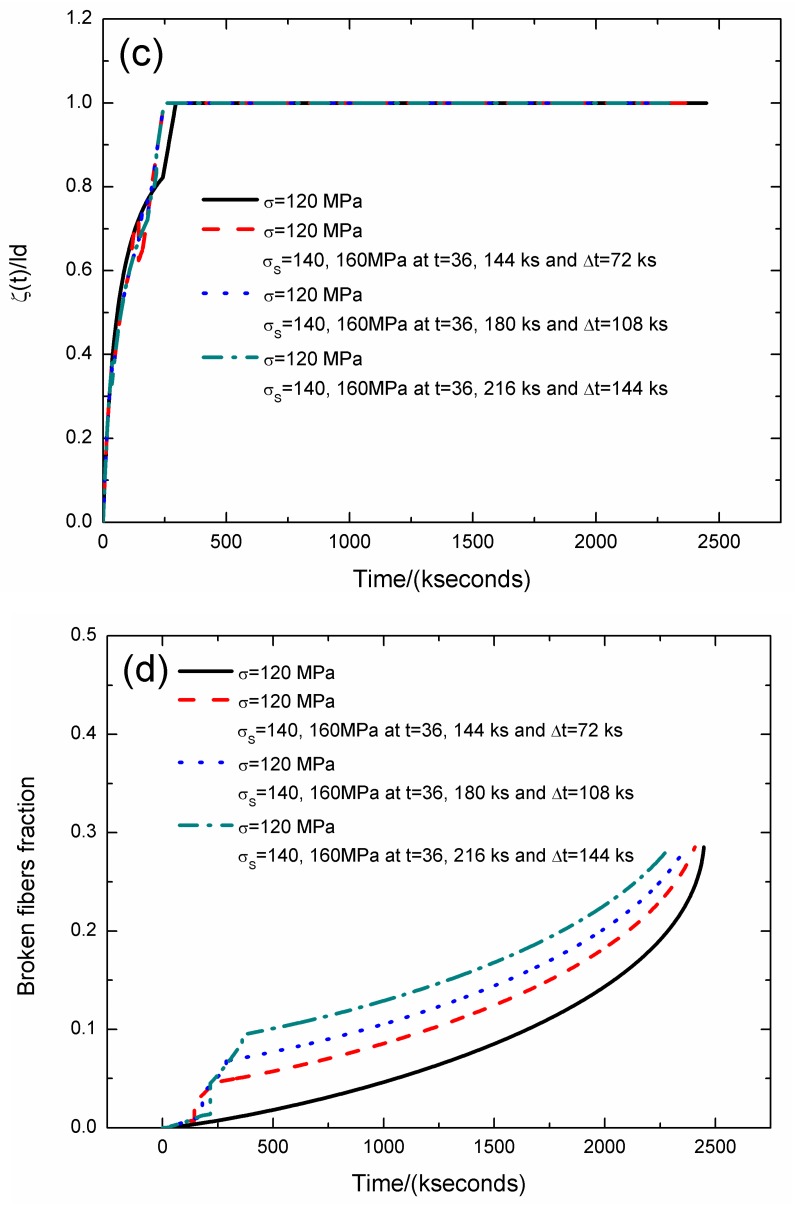
(**a**) The strain versus the time curves; (**b**) the fiber/matrix interface debonding length versus the time curves; (**c**) the fiber/matrix interface oxidation length versus the time curves; and (**d**) the broken fibers fraction versus the time curves of SiC/SiC composite under stress-rupture loading of constant stress of *σ* = 120 MPa, *σ*_S_ = 140/160 MPa at *t* = 36/144, 36/180, 36/216 kseconds and Δt = 72, 108, 144 kseconds at 800 °C in air atmosphere.

The strain, fiber/matrix interface debonding and oxidation length, and the broken fibers fraction of SiC/SiC composite under stress-rupture loading of constant stress of *σ* = 120 MPa, *σ*_S_ = 140/160 MPa at *t* = 36/144, 36/180, 36/216 kseconds and Δ*t* = 72, 108, 144 kseconds at 800 °C in air atmosphere are shown in Figure 8 and Table 6. When the stochastic loading time spacing increases, the stress-rupture lifetime decreases, and the time for the interface complete debonding increases.

When the stochastic loading time spacing is Δ*t* = 72 kseconds, the stress-rupture lifetime is *t* = 2407.2 kseconds; the time for the interface complete debonding is *t* = 160 kseconds at stochastic stress of *σ*_S_ = 160 MPa; the time for the interface complete oxidation is *t* = 246.8 kseconds at constant stress of *σ* = 120 MPa; the failure strain is *ε*_c_ = 0.197%; and the broken fibers fraction is *P* = 0.285. When the stochastic loading time spacing is Δ*t* = 144 kseconds, the stress-rupture lifetime is *t* = 2294.8 kseconds; the time for the interface complete debonding is *t* = 205.7 kseconds at constant stress of *σ* = 120 MPa; the time for the interface complete oxidation is *t* = 246.8 kseconds at stochastic stress of *σ*_S_ = 160 MPa; the failure strain is *ε*_c_ = 0.192%; and the broken fibers fraction is *P* = 0.285.

### 3.3. Case IV

**Figure 9:**
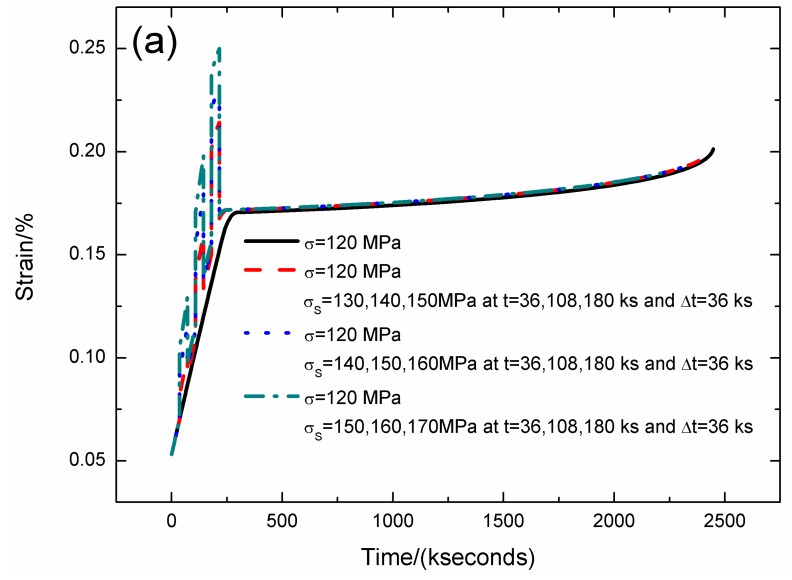

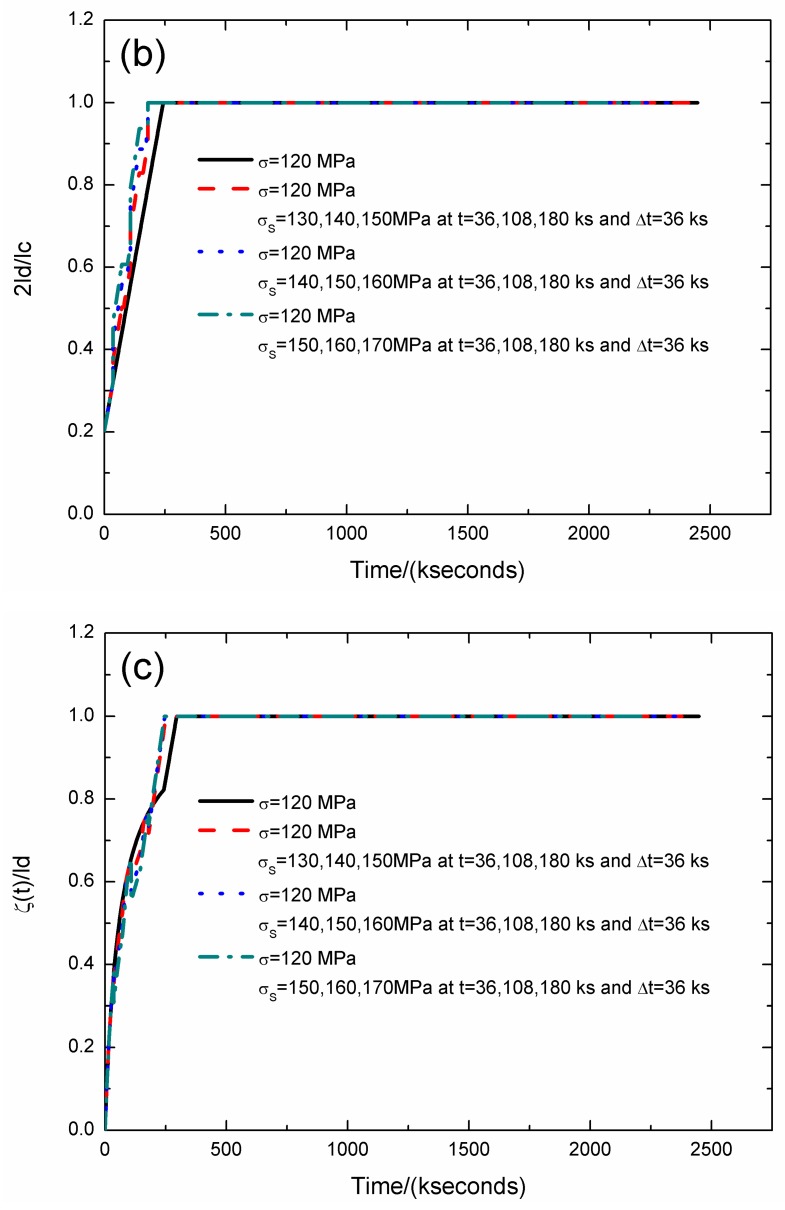

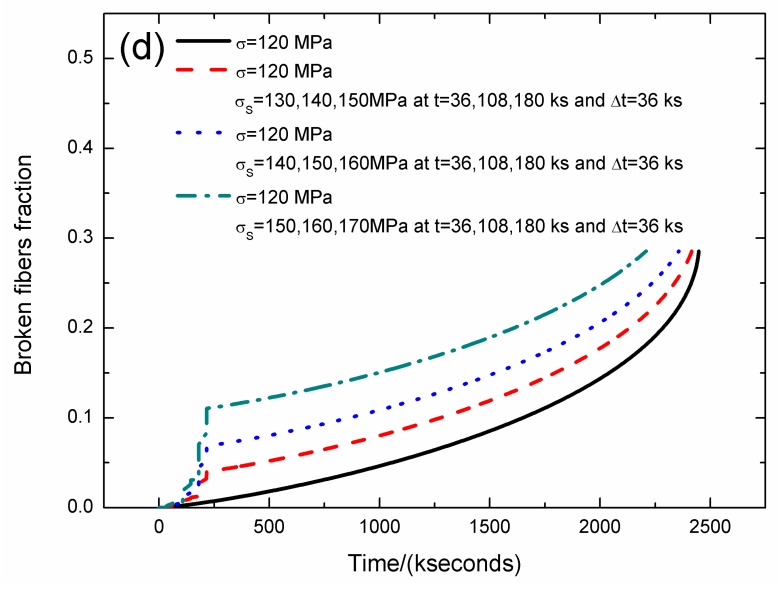
(**a**) The strain versus the time curves; (**b**) the fiber/matrix interface debonding length versus the time curves; (**c**) the fiber/matrix interface oxidation length versus the time curves; and (**d**) the broken fibers fraction versus the time curves of SiC/SiC composite under stress-rupture loading of constant stress of *σ* = 120 MPa, *σ*_S_ = 130/140/150, 140/150/160, 150/160/170 MPa at *t* = 36/108/180 kseconds and Δ*t* = 36 kseconds at 800 °C in air atmosphere.

For the stochastic loading of Case IV, the strain, fiber/matrix interface debonding and oxidation length, and the broken fibers fraction of SiC/SiC composite under stress-rupture loading of constant stress of *σ* = 120 MPa, *σ*_S_ = 130/140/150, 140/150/160, 150/160/170 MPa at *t* = 36/108/180 kseconds and Δ*t* = 36 kseconds at 800 °C in air atmosphere are shown in Figure 9 and Table 7. When the stochastic loading stress increases, the stress-rupture lifetime decreases, and the time for the interface complete oxidation decreases.

When the stochastic loading stress is *σ*_S_ = 130, 140, 150 MPa, the stress-rupture lifetime is *t* = 2416.2 kseconds; the time for the interface complete debonding is *t* = 180 kseconds at stochastic loading stress of *σ*_S_ = 150 MPa; the time for the interface complete oxidation is *t* = 251.3 kseconds at constant stress of *σ* = 120 MPa; the failure strain is *ε*_c_ = 0.198%; and the broken fibers fraction is *P* = 0.285. When the stochastic loading stress is *σ*_S_ = 140, 150, 160 MPa, the stress-rupture lifetime is *t* = 2357.2 kseconds; the time for the interface complete debonding is *t* = 180 kseconds at stochastic loading stress of *σ*_S_ = 160 MPa; the time for the interface complete oxidation is *t* = 246.8 kseconds at constant stress of *σ* = 120 MPa; the failure strain is *ε*_c_ = 0.194%; and the broken fibers fraction is *P* = 0.285. When the stochastic loading stress is *σ*_S_ = 150, 160, 170 MPa, the stress-rupture lifetime is *t* = 2209.1 kseconds; the time for the interface complete debonding is *t* = 180 kseconds at stochastic loading stress of *σ*_S_ = 170 MPa; the time for the interface complete oxidation is *t* = 244.4 kseconds at constant stress of *σ* = 120 MPa; the failure strain is *ε*_c_ = 0.189%; and the broken fibers fraction is *P* = 0.285.

**Figure 10:**
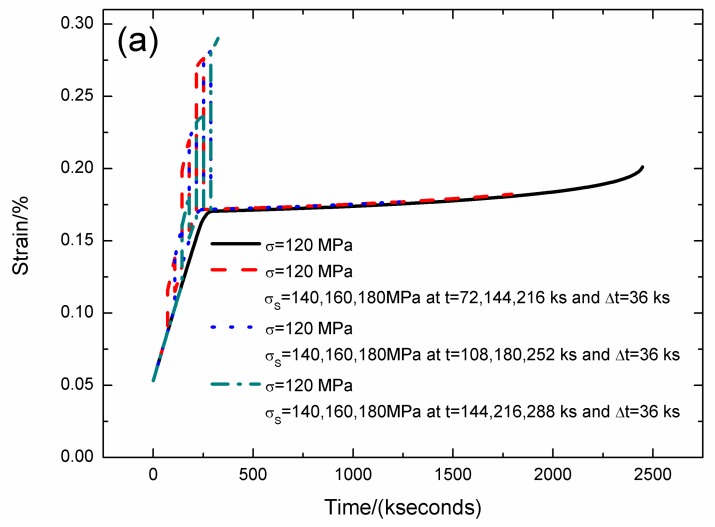

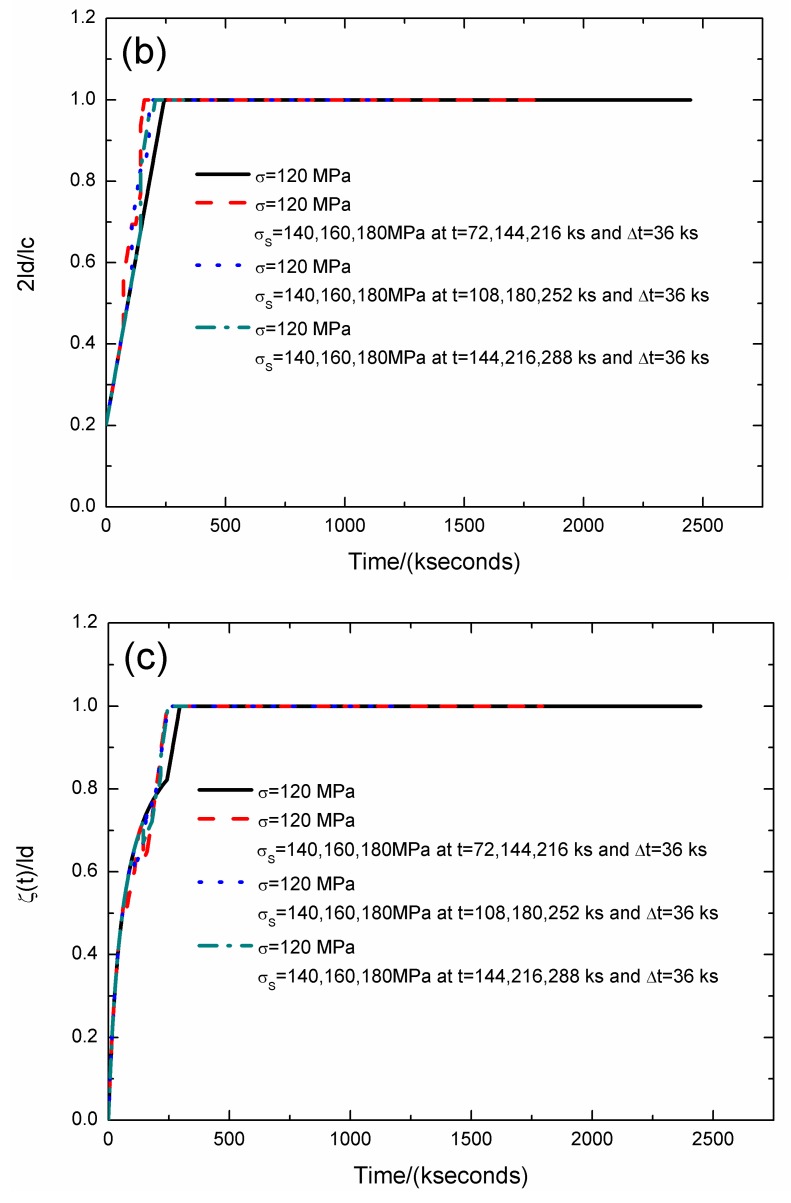

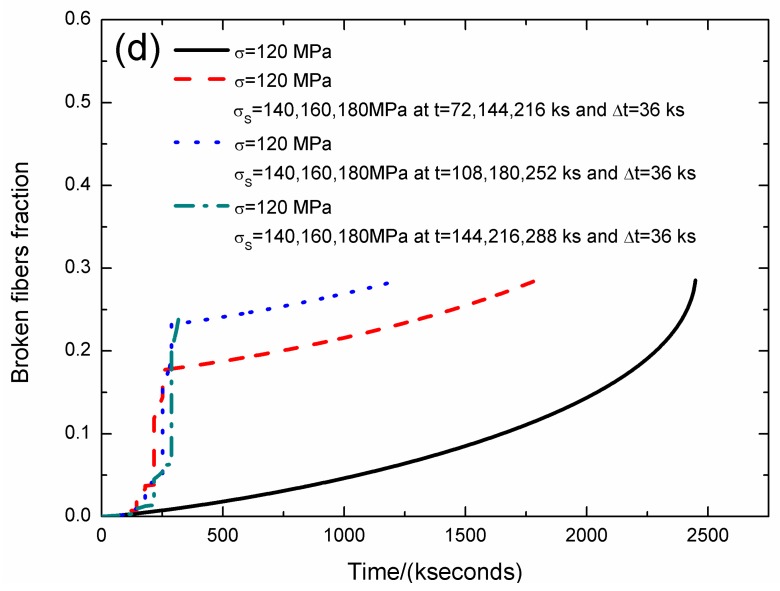
(**a**) The strain versus the time curves; (**b**) the fiber/matrix interface debonding length versus the time curves; (**c**) the fiber/matrix interface oxidation length versus the time curves; and (**d**) the broken fibers fraction versus the time curves of SiC/SiC composite under stress-rupture loading of constant stress of *σ* = 120 MPa, *σ*_S_ = 140/160/180 MPa at *t* = 72/144/216 kseconds and Δt = 36 kseconds at 800 °C in air atmosphere.

The strain, fiber/matrix interface debonding and oxidation length, and the broken fibers fraction of SiC/SiC composite under stress-rupture loading of constant stress of *σ* = 120 MPa, *σ*_S_ = 140/160/180 MPa at *t* = 72/144/216, 108/180/252, 144/216/288 kseconds and Δ*t* = 36 kseconds at 800 °C in air atmosphere are shown in Figure 10 and Table 8. When the stochastic loading time increases, the stress-rupture lifetime decreases, and the time for the interface complete debonding and oxidation increases.

When the stochastic loading time is *t* = 72, 144, 216 kseconds, the stress-rupture lifetime is *t* = 1794 kseconds; the time for the interface complete debonding is *t* = 160 kseconds at stochastic loading stress of *σ*_S_ = 160 MPa; the time for the interface complete oxidation is *t* = 243.3 kseconds at stochastic loading stress of *σ*_S_ = 180 MPa; the failure strain is *ε*_c_ = 0.182%; and the broken fibers fraction is *P* = 0.285. When the stochastic loading time is *t* = 144, 216, 288 kseconds, the stress-rupture lifetime is *t* = 324 kseconds; the time for the interface complete debonding is *t* = 205.7 kseconds at constant stress of *σ* = 120 MPa; the time for the interface complete oxidation is *t* = 246.8 kseconds at stochastic loading stress of *σ*_S_ = 160 MPa; the failure strain is *ε*_c_ = 0.29%; and the broken fibers fraction is *P* = 0.25.

**Figure 11:**
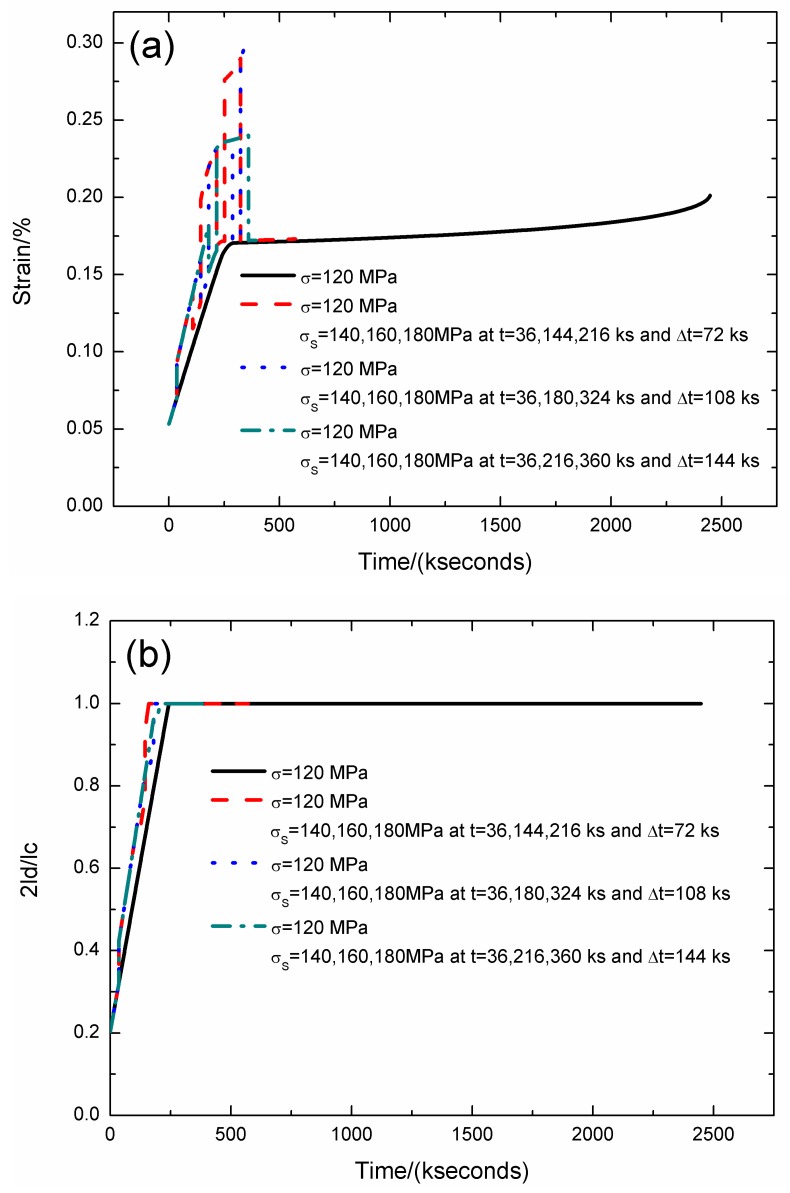

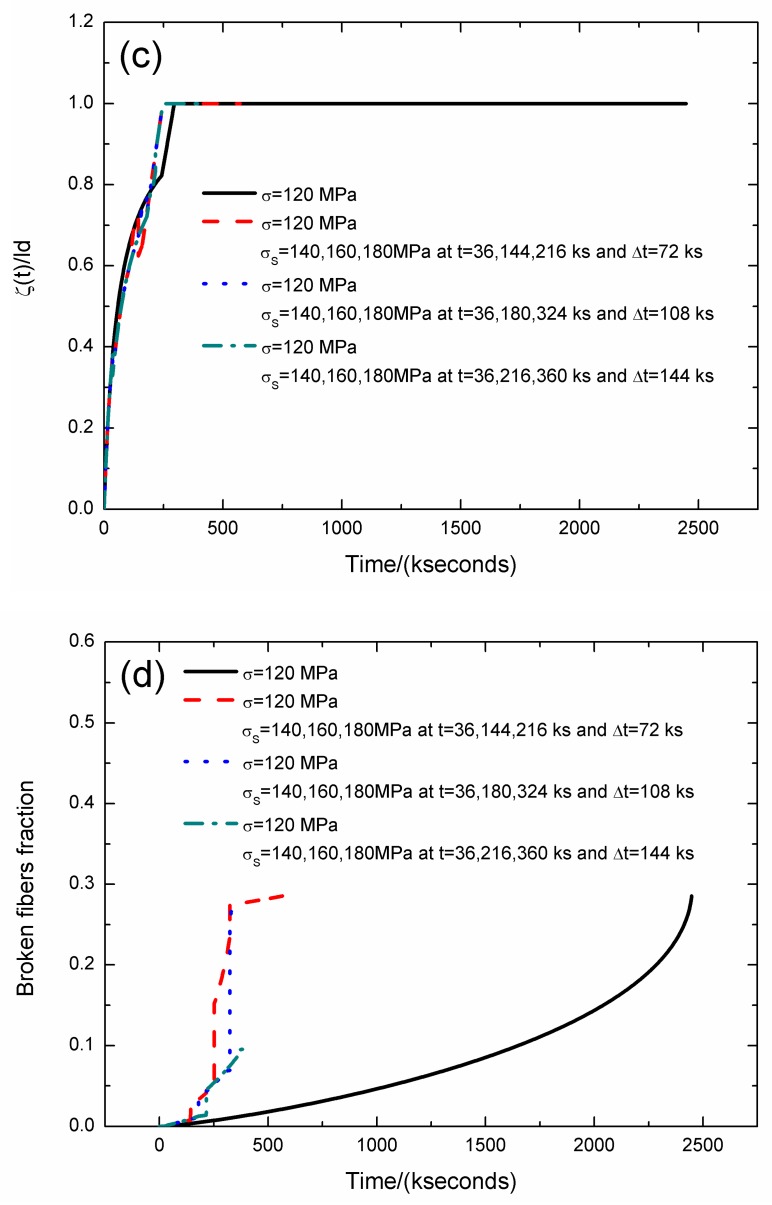
(**a**) The strain versus the time curves; (**b**) the fiber/matrix interface debonding length versus the time curves; (**c**) the fiber/matrix interface oxidation length versus the time curves; and (**d**) the broken fibers fraction versus the time curves of SiC/SiC composite under stress-rupture loading of constant stress of *σ* = 120 MPa, *σ*_S_ = 140/160/180 MPa at *t* = 36/144/216, 36/180/324, 36/216/360 kseconds and Δt = 72, 108, 144 kseconds at 800 °C in air atmosphere.

The strain, fiber/matrix interface debonding and oxidation length, and the broken fibers fraction of SiC/SiC composite under stress-rupture loading of constant stress of *σ* = 120 MPa, *σ*_S_ = 140/160/180 MPa at *t* = 36/144/216, 36/180/324, 36/216/360 kseconds and Δ*t* = 72, 108, 144 kseconds at 800 °C in air atmosphere are shown in Figure 11 and Table 9. When the stochastic loading time spacing increases, the stress-rupture lifetime decreases, and the time for the interface complete debonding increases.

When the stochastic loading time spacing is Δ*t* = 72 kseconds, the stress-rupture lifetime is *t* = 572.4 kseconds; the time for the interface complete debonding is *t* = 160 kseconds at stochastic loading stress of *σ*_S_ = 160 MPa; the time for the interface complete oxidation is *t* = 246.8 kseconds at constant stress of *σ* = 120 MPa; the failure strain is *ε*_c_ = 0.173%; and the broken fibers fraction is *P* = 0.285. When the stochastic loading time spacing is Δ*t* = 144 kseconds, the stress-rupture lifetime is *t* = 396 kseconds; the time for the interface complete debonding is *t* = 205.7 kseconds at constant stress of *σ* = 120 MPa; the time for the interface complete oxidation is *t* = 246.8 kseconds at stochastic loading stress of *σ*_S_ = 160 MPa.

## 4. Conclusions

In this paper, the damage evolution and lifetime of fiber-reinforced CMCs under stress-rupture with stochastic loading at intermediate temperatures were investigated. The relationships between the stochastic loading stress level, time, time spacing, damage mechanisms of matrix cracking, interface debonding and oxidation, and fiber failure were established. The strain, fiber/matrix interface debonding and oxidation length, and the broken fibers fraction versus the time curves of SiC/SiC composite under constant stress and three different stochastic loading conditions were analyzed. The effects of the stochastic loading stress level, stochastic loading time, and time spacing on the damage evolution and lifetime of SiC/SiC composite were discussed. For the stochastic loading of Cases II, III, and IV, the stress-rupture lifetime decreases with increasing stochastic loading stress level, time, and time spacing. The time for the interface complete debonding and oxidation is affected by the loading mode, stress level, loading time, and time spacing.

## Figures and Tables

**Table 1:** The strain, fiber/matrix interface debonding and oxidation length, and broken fibers fraction of SiC/SiC composite under stress-rupture loading of constant stress of *σ* = 120 MPa, *σ*_S_ = 140, 160, 180 MPa at *t* = 36 kseconds and Δt = 36 kseconds at 800 °C in air atmosphere.

*σ*/MPa	120	120	120	120	120			
*t*/kseconds	0	36	242.7	295.3	2447.9			
*ε*_c_/%	0.053	0.07	0.163	0.17	0.201			
2*l*_d_/*l*_c_	0.203	0.322	1.0	1.0	1.0			
*ζ*/*l*_d_	0	0.380	0.822	1.0	1.0			
*P*	1 × 10^−6^	6.5 × 10^−4^	0.007	0.009	0.285			
*σ*/MPa	120	120	140	140	120	120	120	120
*t*/kseconds	0	36	36	72	72	205.7	259.4	2446.9
*ε*_c_/%	0.053	0.07	0.093	0.115	0.096	0.162	0.171	0.202
2*l*_d_/*l*_c_	0.203	0.322	0.423	0.558	0.558	1.0	1.0	1.0
*ζ*/*l*_d_	0	0.380	0.328	0.498	0.498	0.794	1.0	1.0
*P*	1 × 10^−6^	6.5 × 10^−4^	0.001	0.004	0.004	0.008	0.01	0.285
*σ*/MPa	120	120	160	160	120	120	200	200
*t*/kseconds	0	36	36	72	72	192.6	246.8	2444.3
*ε*_c_/%	0.053	0.07	0.116	0.143	0.099	0.162	0.171	0.201
2*l*_d_/*l*_c_	0.203	0.322	0.506	0.649	0.649	1.0	1.0	1.0
*ζ*/*l*_d_	0	0.380	0.289	0.45	0.45	0.781	1.0	1.0
*P*	1 × 10^−6^	6.5 × 10^−4^	0.003	0.008	0.008	0.012	0.014	0.285
*σ*/MPa	120	120	180	180	120	120	120	120
*t*/kseconds	0	36	36	72	72	189.1	243.1	2437
*ε*_c_/%	0.053	0.07	0.138	0.17	0.098	0.162	0.171	0.2
2*l*_d_/*l*_c_	0.203	0.322	0.578	0.724	0.724	1.0	1.0	1.0
*ζ*/*l*_d_	0	0.380	0.257	0.409	0.409	0.778	1.0	1.0
*P*	1 × 10^−6^	6.5 × 10^−4^	0.007	0.018	0.018	0.022	0.024	0.285

**Table 2:** The strain, fiber/matrix interface debonding and oxidation length, and broken fibers fraction of SiC/SiC composite under stress-rupture loading of constant stress of *σ* = 120 MPa, *σ*_S_ = 140 MPa at *t* = 72, 108, 144 kseconds and Δt = 36 kseconds at 800 °C in air atmosphere.

*σ*/MPa	120	120	120	120	120			
*t*/kseconds	0	36	242.7	295.3	2447.9			
*ε*_c_/%	0.053	0.07	0.163	0.17	0.201			
2*l*_d_/*l*_c_	0.203	0.322	1.0	1.0	1.0			
*ζ*/*l*_d_	0	0.380	0.822	1.0	1.0			
*P*	1 × 10^−6^	6.5 × 10^−4^	0.007	0.009	0.285			
*σ*/MPa	120	120	140	140	120	120	120	120
*t*/kseconds	0	72	72	108	108	205.7	259.4	2446.2
*ε*_c_/%	0.053	0.087	0.115	0.138	0.115	0.162	0.171	0.201
2*l*_d_/*l*_c_	0.203	0.44	0.558	0.694	0.694	1.0	1.0	1.0
*ζ*/*l*_d_	0	0.555	0.498	0.601	0.601	0.794	1.0	1.0
*P*	1 × 10^−6^	0.0015	0.0039	0.0065	0.0065	0.0097	0.011	0.285
*σ*/MPa	120	120	140	140	120	120	200	200
*t*/kseconds	0	108	108	144	144	205.7	259.4	2445.2
*ε*_c_/%	0.053	0.103	0.138	0.16	0.133	0.162	0.171	0.201
2*l*_d_/*l*_c_	0.203	0.558	0.694	0.829	0.829	1.0	1.0	1.0
*ζ*/*l*_d_	0	0.656	0.601	0.671	0.671	0.793	1.0	1.0
*P*	1 × 10^−6^	0.002	0.006	0.009	0.009	0.011	0.013	0.285
*σ*/MPa	120	120	140	140	120	120	120	120
*t*/kseconds	0	144	144	180	180	205.7	259.4	2444.1
*ε*_c_/%	0.053	0.12	0.16	0.182	0.152	0.162	0.171	0.201
2*l*_d_/*l*_c_	0.203	0.677	0.829	0.964	0.964	1.0	1.0	1.0
*ζ*/*l*_d_	0	0.722	0.671	0.72	0.72	0.793	1.0	1.0
*P*	1 × 10^−6^	0.003	0.009	0.012	0.012	0.013	0.015	0.285

**Table 3:** The strain, fiber/matrix interface debonding and oxidation length, and broken fibers fraction of SiC/SiC composite under stress-rupture loading of constant stress of *σ* = 120 MPa, *σ*_S_ = 140 MPa at *t* = 36 kseconds and Δt = 72, 108, 144kseconds at 800 °C in air atmosphere.

*σ*/MPa	120	120	120	120	120			
*t*/kseconds	0	36	242.7	295.3	2447.9			
*ε*_c_/%	0.053	0.07	0.163	0.17	0.201			
2*l*_d_/*l*_c_	0.203	0.322	1.0	1.0	1.0			
*ζ*/*l*_d_	0	0.380	0.822	1.0	1.0			
*P*	1 × 10^−6^	6.5 × 10^−4^	0.007	0.009	0.285			
*σ*/MPa	120	120	140	140	120	120	120	120
*t*/kseconds	0	36	36	108	108	205.7	259.4	2446.2
*ε*_c_/%	0.053	0.07	0.093	0.138	0.115	0.162	0.171	0.201
2*l*_d_/*l*_c_	0.203	0.322	0.423	0.694	0.694	1.0	1.0	1.0
*ζ*/*l*_d_	0	0.380	0.328	0.601	0.601	0.794	1.0	1.0
*P*	1 × 10^−6^	6.5 × 10^−4^	0.001	0.006	0.006	0.009	0.012	0.285
*σ*/MPa	120	120	140	140	120	120	200	200
*t*/kseconds	0	36	36	144	144	205.7	259.4	2445.2
*ε*_c_/%	0.053	0.07	0.093	0.160	0.133	0.162	0.171	0.201
2*l*_d_/*l*_c_	0.203	0.322	0.423	0.829	0.829	1.0	1.0	1.0
*ζ*/*l*_d_	0	0.380	0.328	0.671	0.671	0.793	1.0	1.0
*P*	1 × 10^−6^	6.5 × 10^−4^	0.001	0.009	0.009	0.011	0.013	0.285
*σ*/MPa	120	120	140	140	120	120	120	120
*t*/kseconds	0	36	36	180	180	205.7	259.4	2444.1
*ε*_c_/%	0.053	0.07	0.093	0.182	0.152	0.162	0.171	0.201
2*l*_d_/*l*_c_	0.203	0.322	0.423	0.964	0.964	1.0	1.0	1.0
*ζ*/*l*_d_	0	0.380	0.328	0.72	0.72	0.794	1.0	1.0
*P*	1 × 10^−6^	6.5 × 10^−4^	0.001	0.012	0.012	0.013	0.015	0.285

**Table 4:** The strain, fiber/matrix interface debonding and oxidation length, and broken fibers fraction of SiC/SiC composite under stress-rupture loading of constant stress of *σ* = 120 MPa, *σ*_S_ = 130/140, 140/150, 150/160 MPa at *t* = 36/108 kseconds and Δt = 36 kseconds at 800 °C in air atmosphere.

*σ*/MPa	120	120	120	120	120							
*t*/kseconds	0	36	242.7	295.3	2447.9							
*ε*_c_/%	0.053	0.07	0.163	0.17	0.201							
2*l*_d_/*l*_c_	0.203	0.322	1.0	1.0	1.0							
*ζ*/*l*_d_	0	0.380	0.822	1.0	1.0							
*P*	1 × 10^−6^	6.5 × 10^−4^	0.007	0.009	0.285							
*σ*/MPa	120	120	130	130	120	120	140	140	120	120	120	120
*t*/kseconds	0	36	36	72	72	108	108	144	144	205.7	259.4	2444.1
*ε*_c_/%	0.053	0.07	0.082	0.101	0.092	0.109	0.138	0.16	0.133	0.162	0.171	0.2
2*l*_d_/*l*_c_	0.203	0.322	0.375	0.503	0.503	0.603	0.694	0.829	0.829	1.0	1.0	1.0
*ζ*/*l*_d_	0	0.380	0.352	0.525	0.525	0.656	0.602	0.671	0.671	0.794	1.0	1.0
*P*	1 × 10^−6^	6.5 × 10^−4^	0.001	0.0025	0.0025	0.0035	0.0074	0.0103	0.0113	0.013	0.015	0.285
*σ*/MPa	120	120	140	140	120	120	150	150	120	120	120	120
*t*/kseconds	0	36	36	72	72	108	108	144	144	197.3	251.3	2438.1
*ε*_c_/%	0.053	0.07	0.093	0.115	0.096	0.113	0.154	0.179	0.137	0.162	0.171	0.2
2*l*_d_/*l*_c_	0.203	0.322	0.423	0.558	0.558	0.635	0.747	0.887	0.887	1.0	1.0	1.0
*ζ*/*l*_d_	0	0.380	0.328	0.498	0.498	0.656	0.576	0.647	0.647	0.786	1.0	1.0
*P*	1 × 10^−6^	6.5 × 10^−4^	0.0016	0.0039	0.0039	0.0049	0.0123	0.0168	0.0192	0.021	0.023	0.285
*σ*/MPa	120	120	150	150	120	120	160	160	120	120	120	120
*t*/kseconds	0	36	36	72	72	108	108	144	144	192.6	246.8	2425.9
*ε*_c_/%	0.053	0.07	0.104	0.129	0.098	0.116	0.170	0.198	0.138	0.162	0.171	0.199
2*l*_d_/*l*_c_	0.203	0.322	0.467	0.607	0.607	0.656	0.792	0.936	0.936	1.0	1.0	1.0
*ζ*/*l*_d_	0	0.380	0.307	0.473	0.473	0.656	0.553	0.624	0.624	0.781	1.0	1.0
*P*	1 × 10^−6^	6.5 × 10^−4^	0.002	0.0059	0.0059	0.0069	0.019	0.0269	0.0305	0.0324	0.034	0.285

**Table 5:** The strain, fiber/matrix interface debonding and oxidation length, and broken fibers fraction of SiC/SiC composite under stress-rupture loading of constant stress of *σ* = 120 MPa, *σ*_S_ = 140/160 MPa at *t* = 72/144, 108/180, 144/216 kseconds and Δt = 36 kseconds at 800 °C in air atmosphere.

*σ*/MPa	120	120	120	120	120							
*t*/kseconds	0	36	242.7	295.3	2447.9							
*ε*_c_/%	0.053	0.07	0.163	0.17	0.201							
2*l*_d_/*l*_c_	0.203	0.322	1.0	1.0	1.0							
*ζ*/*l*_d_	0	0.380	0.822	1.0	1.0							
*P*	1 × 10^−6^	6.5 × 10^−4^	0.007	0.009	0.285							
*σ*/MPa	120	120	140	140	120	120	160	160	160	120	120	120
*t*/kseconds	0	72	72	108	108	144	144	160	180	180	246.8	2418.4
*ε*_c_/%	0.053	0.087	0.115	0.138	0.114	0.132	0.198	0.209	0.219	0.157	0.171	0.198
2*l*_d_/*l*_c_	0.203	0.44	0.558	0.694	0.694	0.77	0.936	1.0	1.0	1.0	1.0	1.0
*ζ*/*l*_d_	0	0.556	0.498	0.601	0.601	0.722	0.624	0.649	0.73	0.73	1.0	1.0
*P*	1 × 10^−6^	0.0015	0.0039	0.00654	0.00654	0.00766	0.025	0.029	0.033	0.037	0.039	0.285
*σ*/MPa	120	120	140	140	120	120	160	160	120	120	120	
*t*/kseconds	0	108	108	144	144	180	180	216	216	246.8	2400.9	
*ε*_c_/%	0.053	0.103	0.138	0.16	0.133	0.15	0.219	0.231	0.168	0.171	0.197	
2*l*_d_/*l*_c_	0.203	0.558	0.694	0.829	0.829	0.904	1.0	1.0	1.0	1.0	1.0	
*ζ*/*l*_d_	0	0.656	0.601	0.671	0.671	0.768	0.73	0.876	0.876	1.0	1.0	
*P*	1 × 10^−6^	0.0025	0.0065	0.009	0.009	0.01	0.035	0.043	0.048	0.05	0.285	
*σ*/MPa	120	120	140	140	120	120	160	160	120	120		
*t*/kseconds	0	144	144	180	180	216	216	252	252	2376.8		
*ε*_c_/%	0.053	0.16	0.16	0.182	0.152	0.165	0.231	0.236	0.171	0.195		
2*l*_d_/*l*_c_	0.203	0.676	0.829	0.964	0.964	1.0	1.0	1.0	1.0	1.0		
*ζ*/*l*_d_	0	0.722	0.671	0.72	0.72	0.833	0.876	1.0	1.0	1.0		
*P*	1 × 10^−6^	0.0036	0.0094	0.012	0.012	0.013	0.045	0.054	0.061	0.285		

**Table 6:** The strain, fiber/matrix interface debonding and oxidation length, and broken fibers fraction of SiC/SiC composite under stress-rupture loading of constant stress of *σ* = 120 MPa, *σ*_S_ = 140/160 MPa at *t* = 36/144, 36/180, 36/216 kseconds and Δt = 72, 108, 144 kseconds at 800 °C in air atmosphere.

*σ*/MPa	120	120	120	120	120						
*t*/kseconds	0	36	242.7	295.3	2447.9						
*ε*_c_/%	0.053	0.07	0.163	0.17	0.201						
2*l*_d_/*l*_c_	0.203	0.322	1.0	1.0	1.0						
*ζ*/*l*_d_	0	0.380	0.822	1.0	1.0						
*P*	1 × 10^−6^	6.5 × 10^−4^	0.007	0.009	0.285						
*σ*/MPa	120	120	140	140	120	120	160	160	120	120	120
*t*/kseconds	0	36	36	108	108	144	144	216	216	246.8	2407.2
*ε*_c_/%	0.053	0.07	0.093	0.138	0.114	0.132	0.198	0.231	0.168	0.171	0.197
2*l*_d_/*l*_c_	0.203	0.322	0.423	0.694	0.694	0.77	0.936	1.0	1.0	1.0	1.0
*ζ*/*l*_d_	0	0.380	0.328	0.601	0.601	0.72	0.624	0.876	0.876	1.0	1.0
*P*	1 × 10^−6^	6.5 × 10^−4^	0.0016	0.0065	0.0065	0.007	0.025	0.041	0.045	0.046	0.285
*σ*/MPa	120	120	140	140	120	120	160	160	120	120	
*t*/kseconds	0	36	36	144	144	180	180	288	288	2365.7	
*ε*_c_/%	0.053	0.07	0..093	0.16	0.133	0.15	0.219	0.237	0.171	0.194	
2*l*_d_/*l*_c_	0.203	0.322	0.423	0.829	0.829	0.904	1.0	1.0	1.0	1.0	
*ζ*/*l*_d_	0	0.380	0.328	0.671	0.671	0.768	0.73	1.0	1.0	1.0	
*P*	1 × 10^−6^	6.5 ×10^−4^	0.0016	0.009	0.009	0.01	0.035	0.062	0.067	0.285	
*σ*/MPa	120	120	140	140	120	120	160	160	120	120	
*t*/kseconds	0	36	36	180	180	216	216	360	360	2294.8	
*ε*_c_/%	0.053	0.07	0.093	0.182	0.152	0.166	0.231	0.24	0.172	0.192	
2*l*_d_/*l*_c_	0.203	0.322	0.423	0.964	0.964	1.0	1.0	1.0	1.0	1.0	
*ζ*/*l*_d_	0	0.380	0.328	0.72	0.72	0.833	0.876	1.0	1.0	1.0	
*P*	1 × 10^−6^	6.5 × 10^−4^	0.0016	0.012	0.012	0.013	0.045	0.086	0.094	0.285	

**Table 7:** The strain, fiber/matrix interface debonding and oxidation length, and broken fibers fraction of SiC/SiC composite under stress-rupture loading of constant stress of *σ* = 120 MPa, *σ*_S_ = 130/140/150, 140/150/160, 150/160/170 MPa at *t* = 36/108/180 kseconds and Δt = 36 kseconds at 800 °C in air atmosphere.

*σ*/MPa	120	120	120	120	120										
*t*/kseconds	0	36	242.7	295.3	2447.9										
*ε*_c_/%	0.053	0.07	0.163	0.17	0.201										
2*l*_d_/*l*_c_	0.203	0.322	1.0	1.0	1.0										
*ζ*/*l*_d_	0	0.380	0.822	1.0	1.0										
*P*	1 × 10^−6^	6.5 × 10^−4^	0.007	0.009	0.285										
*σ*/MPa	120	120	130	130	120	120	140	140	120	120	150	150	120	120	120
*t*/kseconds	0	36	36	72	72	108	108	144	144	180	180	216	216	251.3	2416.2
*ε*_c_/%	0.053	0.07	0.081	0.101	0.092	0.109	0.138	0.16	0.133	0.15	0.2	0.214	0.167	0.171	0.198
2*l*_d_/*l*_c_	0.203	0.322	0.375	0.503	0.503	0.604	0.694	0.829	0.829	0.904	1.0	1.0	1.0	1.0	1.0
*ζ*/*l*_d_	0	0.380	0.352	0.525	0.525	0.656	0.602	0.671	0.671	0.768	0.717	0.86	0.86	1.0	1.0
*P*	1 × 10^−6^	6.5 × 10^−4^	0.001	0.0025	0.0025	0.003	0.0075	0.0104	0.0113	0.012	0.027	0.03	0.04	0.041	0.285
*σ*/MPa	120	120	140	140	120	120	150	150	120	120	160	160	120	120	120
*t*/kseconds	0	36	36	72	72	108	108	144	144	180	180	216	216	246.8	2357.2
*ε*_c_/%	0.053	0.07	0.093	0.115	0.096	0.113	0.154	0.179	0.137	0.154	0.22	0.231	0.168	0.171	0.194
2*l*_d_/*l*_c_	0.203	0.322	0.423	0.558	0.558	0.635	0.747	0.887	0.887	0.933	1.0	1.0	1.0	1.0	1.0
*ζ*/*l*_d_	0	0.380	0.328	0.498	0.498	0.656	0.576	0.647	0.647	0.768	0.73	0.876	0.876	1.0	1.0
*P*	1 × 10^−6^	6.5 × 10^−4^	0.0016	0.0039	0.0039	0.005	0.0123	0.0168	0.0192	0.02	0.044	0.053	0.068	0.069	0.285
*σ*/MPa	120	120	150	150	120	120	160	160	120	120	170	170	120	120	120
*t*/kseconds	0	36	36	72	72	108	108	144	144	180	180	216	216	244.4	2209.1
*ε*_c_/%	0.053	0.07	0.104	0.129	0.098	0.116	0.17	0.198	0.138	0.156	0.237	0.25	0.169	0.171	0.189
2*l*_d_/*l*_c_	0.203	0.322	0.467	0.607	0.607	0.656	0.792	0.936	0.936	0.95	1.0	1.0	1.0	1.0	1.0
*ζ*/*l*_d_	0	0.380	0.308	0.473	0.473	0.656	0.553	0.624	0.224	0.768	0.737	0.884	0.884	1.0	1.0
*P*	1 × 10^−6^	6.5 × 10^−4^	0.0024	0.0059	0.0059	0.007	0.019	0.026	0.0305	0.03	0.07	0.083	0.11	0.11	0.285

**Table 8:** The strain, fiber/matrix interface debonding and oxidation length, and broken fibers fraction of SiC/SiC composite under stress-rupture loading of constant stress of *σ* = 120 MPa, *σ*_S_ = 140/160/180 MPa at *t* = 72/144/216, 108/180/252, 144/216/288 kseconds and Δt = 36 kseconds at 800 °C in air atmosphere.

*σ*/MPa	120	120	120	120	120									
*t*/kseconds	0	36	242.7	295.3	2447.9									
*ε*_c_/%	0.053	0.07	0.163	0.17	0.201									
2*l*_d_/*l*_c_	0.203	0.322	1.0	1.0	1.0									
*ζ*/*l*_d_	0	0.380	0.822	1.0	1.0									
*P*	1 × 10^−6^	6.5 × 10^−4^	0.007	0.009	0.285									
*σ*/MPa	120	120	140	140	120	120	160	160	120	120	180	180	120	120
*t*/kseconds	0	72	72	108	108	144	144	180	180	216	216	252	252	1794
*ε*_c_/%	0.053	0.087	0.115	0.138	0.115	0.132	0.198	0.22	0.157	0.168	0.269	0.276	0.171	0.182
2*l*_d_/*l*_c_	0.203	0.44	0.558	0.694	0.694	0.77	0.936	1.0	1.0	1.0	1.0	1.0	1.0	1.0
*ζ*/*l*_d_	0	0.555	0.498	0.601	0.601	0.722	0.624	0.73	0.73	0.876	0.888	1.0	1.0	1.0
*P*	1 × 10^−6^	0.0015	0.0039	0.0065	0.0065	0.0076	0.025	0.033	0.037	0.038	0.118	0.144	0.177	0.285
*σ*/MPa	120	120	140	140	120	120	160	160	120	120	180	180	120	120
*t*/kseconds	0	108	108	144	144	180	180	216	216	252	252	288	288	1230.8
*ε*_c_/%	0.053	0.103	0.138	0.16	0.133	0.15	0.22	0.232	0.168	0.171	0.276	0.281	0.172	0.177
2*l*_d_/*l*_c_	0.203	0.558	0.694	0.829	0.829	0.904	1.0	1.0	1.0	1.0	1.0	1.0	1.0	1.0
*ζ*/*l*_d_	0	0.656	0.601	0.671	0.671	0.768	0.73	0.876	0.876	1.0	1.0	1.0	1.0	1.0
*P*	1 × 10^−6^	0.0025	0.0065	0.0094	0.0094	0.01	0.034	0.043	0.048	0.05	0.155	0.189	0.231	0.285
*σ*/MPa	120	120	140	140	120	120	160	160	120	120	180	180		
*t*/kseconds	0	144	144	180	180	216	216	252	252	288	288	324		
*ε*_c_/%	0.053	0.12	0.16	0.182	0.152	0.165	0.231	0.236	0.171	0.172	0.282	0.29		
2*l*_d_/*l*_c_	0.203	0.677	0.829	0.964	0.964	1.0	1.0	1.0	1.0	1.0	1.0	1.0		
*ζ*/*l*_d_	0	0.722	0.671	0.72	0.72	0.833	0.876	1.0	1.0	1.0	1.0	1.0		
*P*	1 × 10^−6^	0.0037	0.0094	0.012	0.012	0.013	0.045	0.054	0.061	0.063	0.2	0.25		

**Table 9:** The strain, fiber/matrix interface debonding and oxidation length, and broken fibers fraction of SiC/SiC composite under stress-rupture loading of constant stress of *σ* = 120 MPa, *σ*_S_ = 140/160/180 MPa at *t* = 36/144/216, 36/180/324, 36/216/360 kseconds and Δt = 72, 108, 144 kseconds at 800 °C in air atmosphere.

*σ*/MPa	120	120	120	120	120									
*t*/kseconds	0	36	242.7	295.3	2447.9									
*ε*_c_/%	0.053	0.07	0.163	0.17	0.201									
2*l*_d_/*l*_c_	0.203	0.322	1.0	1.0	1.0									
*ζ*/*l*_d_	0	0.380	0.822	1.0	1.0									
*P*	1 × 10^−6^	6.5 × 10^−4^	0.007	0.009	0.285									
*σ*/MPa	120	120	140	140	120	120	160	160	120	120	180	180	120	120
*t*/kseconds	0	36	36	108	108	144	144	216	216	252	252	324	324	572.4
*ε*_c_/%	0.053	0.07	0.093	0.138	0.115	0.132	0.198	0.231	0.168	0.171	0.267	0.29	0.172	0.173
2*l*_d_/*l*_c_	0.203	0.322	0.423	0.694	0.694	0.77	0.936	1.0	1.0	1.0	1.0	1.0	1.0	1.0
*ζ*/*l*_d_	0	0.380	0.328	0.601	0.601	0.722	0.624	0.876	0.876	1.0	1.0	1.0	1.0	1.0
*P*	1 × 10^−6^	6.5 × 10^−4^	0.0016	0.0065	0.0065	0.007	0.025	0.041	0.045	0.046	0.151	0.235	0.274	0.285
*σ*/MPa	120	120	140	140	120	120	160	160	120	120	180	180		
*t*/kseconds	0	36	36	144	144	180	180	288	288	324	324	337.8		
*ε*_c_/%	0.053	0.07	0.093	0.16	0.133	0.15	0.22	0.237	0.171	0.172	0.29	0.295		
2*l*_d_/*l*_c_	0.203	0.322	0.423	0.829	0.829	0.904	1.0	1.0	1.0	1.0	1.0	1.0		
*ζ*/*l*_d_	0	0.380	0.328	0.671	0.671	0.768	0.73	1.0	1.0	1.0	1.0	1.0		
*P*	1 × 10^−6^	6.5 × 10^−4^	0.0016	0.009	0.009	0.01	0.035	0.062	0.067	0.069	0.254	0.285		
*σ*/MPa	120	120	140	140	120	120	160	160	120	120	180			
*t*/kseconds	0	36	36	180	180	216	216	360	360	396	396			
*ε*_c_/%	0.053	0.07	0.093	0.182	0.152	0.165	0.231	0.24	0.172	0.172				
2*l*_d_/*l*_c_	0.203	0.322	0.423	0.964	0.964	1.0	1.0	1.0	1.0	1.0	—			
*ζ*/*l*_d_	0	0.380	0.328	0.72	0.72	0.833	0.876	1.0	1.0	1.0	—			
*P*	1 × 10^−6^	6.5 × 10^−4^	0.0016	0.012	0.012	0.013	0.045	0.086	0.094	0.096	—			

## References

[1] Naslain R. (2004). Design, Preparation and properties of non-oxide CMCs for application in engines and nuclear reactors: An overview. Compos. Sci. Technol..

[2] DiCarlo J.A., Roode M. (2006). Ceramic Composite Development for Gas Turbine Hot Section Components.

[3] Li L. (2018). Damage, Fracture and Fatigue of Ceramic-Matrix Composites.

[4] Li L. (2019). Thermomechanical Fatigue of Ceramic-Matrix Composites.

[5] Recle E., Godin N., Reynaud P., Fantozzi G. (2017). Fatigue lifetime of ceramic matrix composites at intermediate temperature by acoustic emission. Materials.

[6] Ruggles-Wrenn M.B., Christensen D.T., Chamberlain A.L., Lane J.E., Cook T.S. (2011). Effect of frequency and environment on fatigue behavior of a CVI SiC/SiC ceramic matrix composite at 1200 °C. Compos. Sci. Technol..

[7] Dassios K.G., Aggelis D.G., Kordatos E.Z., Matikas T.E. (2013). Cyclic loading of a SiC-fiber reinforced ceramic matrix composite reveals damage mechanisms and thermal residual stress state. Compos. Part A.

[8] Rebillat F., Low I.M. (2014). Advances in self-healing ceramic matrix composites. Advances in Ceramic Matrix Composites.

[9] Lara-Curzio E. (1997). Stress rupture of Nicalon/SiC continuous fiber ceramic composites in air at 950 °C. J. Am. Ceram. Soc..

[10] Morscher G.N. (1997). Tensile stress rupture of SiC_f_/SiC_m_ minicomposites with carbon and boron nitride interphases at elevated temperatures in air. J. Am. Ceram. Soc..

[11] Verrilli M.J., Opila E.J., Calomino A., Kiser J.D. (2004). Effect of environment on the stress-rupture behavior of a carbon-fiber-reinforced silicon carbide ceramic matrix composites. J. Am. Ceram. Soc..

[12] Hussain A., Calabria-Holley J., Lawrence M., Jiang Y. (2019). Hygrothermal and mechanical characterization of novel hemp shiv based thermal insulation composites. Constr. Build. Mater..

[13] Khosravani M.R., Weihberg K. (2017). Experimental investigations of the environmental effects on stability and integrity of composite sandwich T-joints. Mater. Werkst..

[14] Morscher G.N., Hurst J., Brewer D. (2000). Intermediate-temperature stress rupture of a woven Hi-Nicalon, BN-interphase, SiC-matrix composite in air. J. Am. Ceram. Soc..

[15] Morscher G.N., Cawley J.D. (2002). Intermediate temperature strength degradation in SiC/SiC composites. J. Eur. Ceram. Soc..

[16] Li L. (2017). Damage evolution of cross-ply ceramic-matrix composites under stress-rupture and cyclic loading at elevated temperatures in oxidizing atmosphere. Mater. Sci. Eng. A.

[17] Li L. (2017). Synergistic effects of temperature, oxidation, loading frequency and stress-rupture on damage evolution of cross-ply ceramic matrix composites under cyclic fatigue loading at elevated temperatures in oxidizing atmosphere. Eng. Fract. Mech..

[18] Momon S., Moevus M., Godin N., R’Mili M., Reynaud P., Fantozzi G., Fayolle G. (2010). Acoustic emission and lifetime prediction during static fatigue tests on ceramic-matrix-composite at high temperature under air. Compos. Part A.

[19] Godin N., Reynaud P., Fantozzi G. (2019). Contribution of AE analysis in order to evaluate time to failure of ceramic matrix composites. Eng. Fract. Mech..

[20] Ikarashi T., Ogasawara T., Aoki T. (2019). Effects of cyclic tensile loading on rupture behavior of orthogonal 3-D woven SiC fiber/SiC matrix composites at elevated temperatures in air. J. Eur. Ceram. Soc..

[21] Casas L., Martinez-Esnaola J.M. (2003). Modeling the effect of oxidation on the creep behavior of fiber-reinforced ceramic matrix composites. Acta Mater..

[22] Curtin W.A. (1993). Multiple matrix cracking in brittle matrix composites. Acta Metal. Mater..

[23] Gao Y., Mai Y., Cotterell B. (1988). Fracture of fiber-reinforced materials. J. Appl. Math. Phys..

[24] Curtin W.A. (1991). Theory of mechanical properties of ceramic-matrix composites. J. Am. Ceram. Soc..

[25] Lara-Curzio E. (1999). Analysis of oxidation-assisted stress-rupture of continuous fiber-reinforced ceramic matrix composites at intermediate temperatures. Compos. Part A.

